# An Irreversible Inhibitor of HSP72 that Unexpectedly Targets Lysine‐56

**DOI:** 10.1002/anie.201611907

**Published:** 2017-02-22

**Authors:** Jonathan Pettinger, Yann‐Vaï Le Bihan, Marcella Widya, Rob L. M. van Montfort, Keith Jones, Matthew D. Cheeseman

**Affiliations:** ^1^ Cancer Research UK Cancer Therapeutics UnitThe Institute of Cancer ResearchLondonSW7 3RPUK; ^2^ Division of Structural BiologyThe Institute of Cancer ResearchLondonSW7 3RPUK; ^3^ Proteomics Core FacilityThe Institute of Cancer ResearchLondonSW7 3RPUK

**Keywords:** fluorescence polarization, irreversible inhibitors, mass spectrometry, medicinal chemistry, structural biology

## Abstract

The stress‐inducible molecular chaperone, HSP72, is an important therapeutic target in oncology, but inhibiting this protein with small molecules has proven particularly challenging. Validating HSP72 inhibitors in cells is difficult owing to competition with the high affinity and abundance of its endogenous nucleotide substrates. We hypothesized this could be overcome using a cysteine‐targeted irreversible inhibitor. Using rational design, we adapted a validated 8‐*N*‐benzyladenosine ligand for covalent bond formation and confirmed targeted irreversible inhibition. However, no cysteine in the protein was modified; instead, we demonstrate that lysine‐56 is the key nucleophilic residue. Targeting this lysine could lead to a new design paradigm for HSP72 chemical probes and drugs.

Heat shock 70 kDa protein 1 (HSP72) is a stress‐inducible ATPase molecular chaperone, which stabilizes and refolds substrate proteins to maintain cellular homeostasis.^1^ HSP72 is a well‐established target in oncology, as upregulation is associated with poor clinical outcomes^2^ and drug resistance.^3^

A significant hurdle to cellular activity for nucleotide‐competitive inhibitors of HSP72 is the high affinity for its endogenous nucleotide substrates (ADP, *K*_D_≈110 nm).^4^, ^5^ Irreversible inhibition is an important strategy for proteins with high‐affinity substrates,^6^ with the recent renaissance led by drugs targeting the tyrosine kinase EGFR, which circumvent the increased ATP affinity resulting from the T790M resistance mutation.^7^ Owing to the clear clinical potential that a HSP72 inhibitor could offer and with few cell‐active chemical probes to study the role of HSP72 in cancer, we proposed that a nucleotide‐competitive targeted covalent inhibitor could overcome many of these challenges.

Crucial to the success of targeted covalent inhibitors is their two‐step process for inhibition.^8^ The inhibitor first binds reversibly, forming a non‐covalent complex, before covalent bond formation to give the irreversible complex [Equation (1)]. This process means the reactivity of the electrophilic warhead can be reduced, so the reaction is only fast once the complex has formed.^8^ We hypothesized that the validated nucleotide‐competitive 8‐*N*‐benzyladenosine **1** (Scheme 1),^9^ which is a potent targeted reversible inhibitor [Equation (2)], fulfills these criteria and could be modified for targeted covalent inhibitor design.

(1) $$E+I\overset{K{}_{l}}{\underset{}{↔}}\mathrm{EI}\overset{k{}_{\mathrm{inact}}}{\underset{}{\to }}E-I$$

(2) $$E+I\overset{K{}_{i}}{\underset{}{↔}}\mathrm{EI}$$

(3) $$E+I\overset{k{}_{\mathrm{inact}}}{\underset{}{\to }}E-I$$

**Scheme 1:**
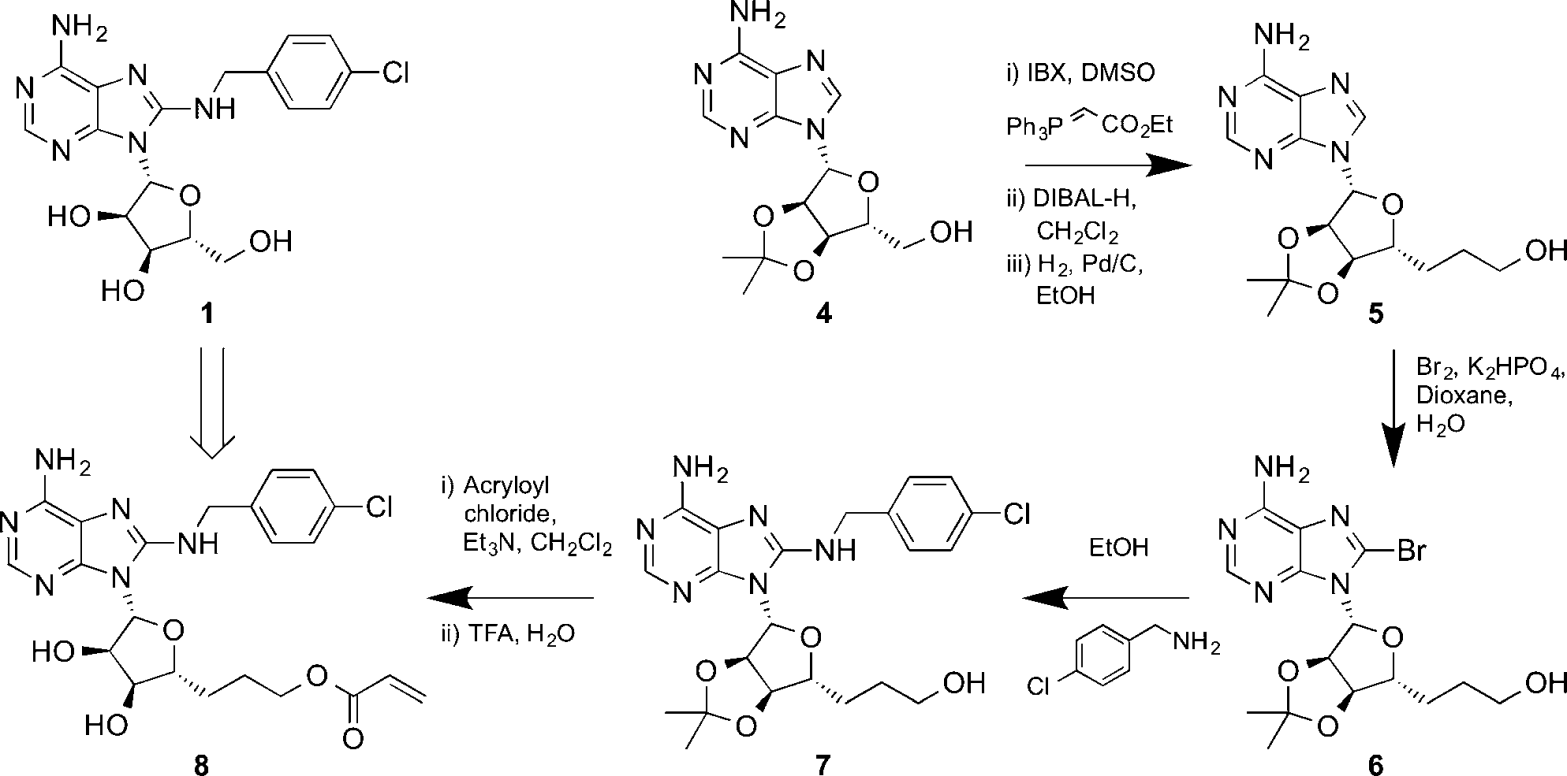
Synthesis of the HSP72‐NBD nucleotide‐competitive targeted covalent inhibitor **8**.

Proteins typically react through solvent‐exposed nucleophilic cysteine residues.^8^ Focusing only on the nucleotide‐binding domain (NBD), analysis of the HSP72 co‐crystal structure, bound with the validated nucleotide‐competitive inhibitor Ver‐155008, (Figure 1, PDB: 4IO8) revealed three residues: Cys17, Cys267 and Cys306 (see the Supporting Information).^4^ Three irreversible inhibitors of HSP70 have been reported; YK5 **2** 10 and oridonin **3** 11 are proposed to target Cys267 of the NBD, while the natural product novolactone^12^ targets Glu444 in the substrate‐binding domain. Cys267 is distal from the validated targeted reversible inhibitor **1** binding site and is buried deeply in a hydrophobic region, requiring significant protein conformational change to become solvent‐exposed, so is incompatible with rational targeted covalent inhibitor design.^13^ Of the remaining reactive cysteine residues, Cys306 is also positioned too far from the binding site. However, Cys17 is at the bottom of the binding cleft with an unhindered vector pointing directly towards the 5′‐position of the reversibly‐bound ligand. We believed Cys17 could potentially be targeted as the key nucleophilic protein residue and that the linker and electrophile could be developed by rational design (Figure 1).

**Figure 1:**
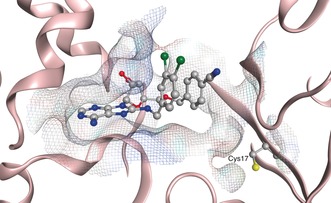
Targeting Cys17 at the base of the targeted reversible inhibitor binding site of HSP72‐NBD (PDB: 4IO8, residues 3–379) only key residues are shown, solvent and hydrogens omitted for clarity, carbon=grey, oxygen=red, nitrogen=blue, chlorine=green, sulfur=yellow.

HSP72 is a highly flexible protein, which complicates inhibitor design.^9^ The distance between Cys17 and the 5′‐position of the nucleoside analogues depends on the protein conformation, ranging from 9.2–10.7 Å (see Supporting Information). Our design strategy required a versatile synthesis of 5′‐adenosine derivatives, so that the linker could span the flexible gap to the nucleophilic residue, while retaining the 8‐*N*‐benzyl moiety to maintain reversible affinity (Figure 2). From analysis of our model, we predicted that a 3‐carbon flexible linker at the 5′‐position would span the distance in the open conformation of the HSP72‐NBD. Typically, the electrophile in a targeted covalent inhibitor is an *N*‐arylacrylamide^14^ but our irreversible inhibitor would require an aliphatic electrophile, so we incorporated an acrylate group to maintain reactivity (Scheme 1).

**Figure 2:**
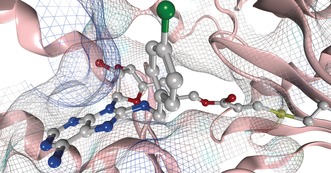
HSP72‐NBD Cys17 irreversible inhibitor model generated using MOE 2013.0801, only key residues are shown, solvent and hydrogens omitted for clarity, carbon=grey, oxygen=red, nitrogen=blue, chlorine=green, sulfur‐=yellow.

Two‐carbon homologation of 3′,4′‐acetonide adenosine **4** gave primary alcohol **5** in 51 % yield over three steps. Selective C8‐bromination then gave the key intermediate **6**, which was treated with *para*‐chlorobenzylamine to give **7** in 89 % yield over two steps. Acylation of the primary alcohol with acryloyl chloride and finally deprotection of the acetonide gave our targeted covalent inhibitor **8** in seven steps and 6 % overall yield. As controls, targeted reversible inhibitor matched‐pair **9**, without the electrophilic warhead, and acetonide‐protected **10**, which was predicted to be a non‐binding but still electrophilic analogue, were synthesized in a similar manner (Figure 3 and the Supporting Information).

**Figure 3:**
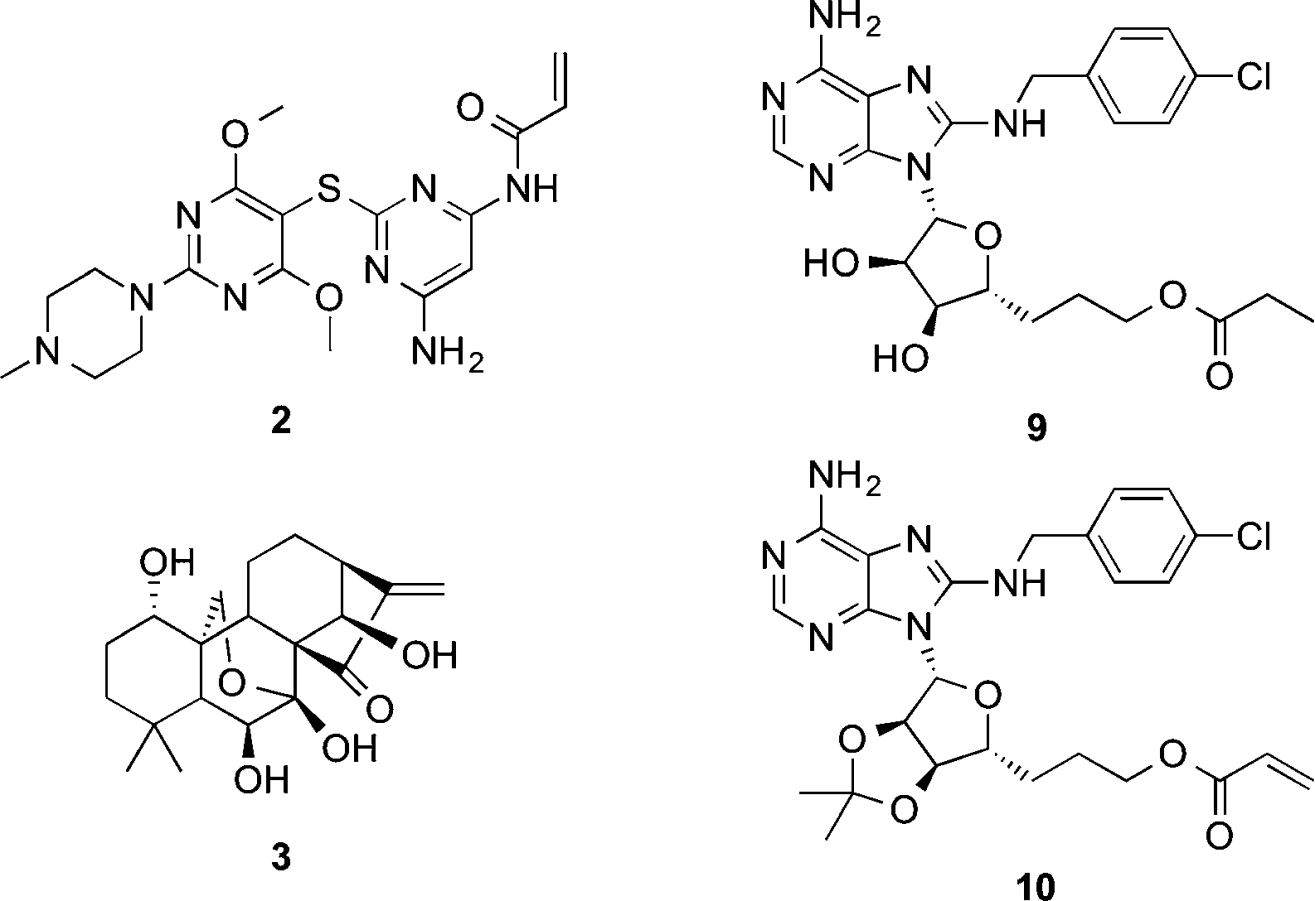
HSP72‐NBD tool compounds and irreversible inhibitor assay controls.

To establish whether our rationally designed targeted covalent inhibitor **8** could form a reversible complex, its affinity was assessed using a fluorescence polarization (FP) assay to measure the inhibition of binding of a fluorescent nucleotide‐probe (Figure 4 and the Supporting Information).^15^ The initial *K*_i_ values for 8‐*N*‐benzyladenosine **1** and the reversible matched pair **9** were 1.9 μm and 42 μm, respectively.^16^ The acetonide‐protected irreversible **10** displayed no affinity, while **8** gave an apparent initial affinity of 17 μm. The time‐dependence of the *K*_i_ value was then assessed for each ligand, as irreversible inhibitors should show apparent increasing affinity over time.^8^ As expected, 8‐*N*‐benzyladenosine **1** and the reversible matched pair **9** displayed no time‐dependence but **8** displayed an increase in its affinity over 46 h, consistent with covalent bond formation (Figure 4). The putative HSP70 irreversible inhibitors, YK5 **2** and oridonin **3**, displayed no initial activity in this assay (200 μm), reflecting their low potency as nucleotide‐competitive targeted reversible inhibitors. YK5 **2** also displayed no time‐dependence in the FP‐assay (22 h), in contrast to oridonin **3**, which despite no initial affinity did show displacement of the probe over time (16 h), consistent with activity as a non‐specific affinity label [Equation (3), see the Supporting Information for details].

**Figure 4:**
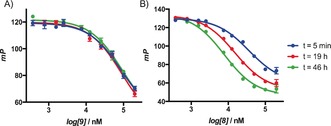
a) Targeted reversible inhibitor **9** shows no time‐dependent displacement of the FP‐probe. b) Time‐dependent inhibition of the HSP72‐NBD with targeted covalent inhibitor **8** assessed using displacement of a nucleotide‐derived FP‐probe; each point is carried out in triplicate, error bars show the arithmetic mean±SEM.

To ensure that the irreversible inhibition observed with **8** was not due to the compound acting as a non‐specific affinity label, the non‐binding irreversible matched pair **10** and the electrophile, *O*‐methyl acrylate, were assayed over the same extended time period but continued to display no activity. However, even though the potent electrophile iodoacetamide displayed no apparent reversible binding, it did displace the FP‐probe over time, presumably due to its greater reactivity compared to *O*‐methyl acrylate (see the Supporting Information).^13^

To confirm the formation of a covalent bond, a solution of **8** (200 μm, ≈12× initial *K*_i_) and HSP72‐NBD (2.3 μm) in tris‐buffer were incubated together and the samples analyzed by intact‐protein mass‐spectrometry (MS, Figure 5). The MS data revealed a time‐dependent increase in a peak at 43 630 Da (corresponding to HSP72‐NBD + 490 Da), consistent with covalent bond formation with **8**, and a minor *bis*‐adduct of HSP72‐NBD + 980 Da. Control compounds **9** and **10** displayed no significant modification over the same period. YK5 **2** displayed no significant modification in this assay and oridonin **3** appeared to react with HSP72‐NBD multiple times (see the Supporting Information).

**Figure 5:**
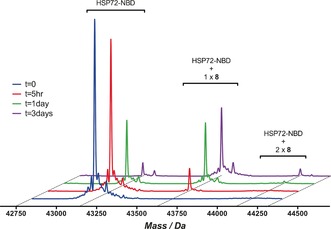
Intact‐protein MS trace for the time‐dependent modification of HSP72‐NBD with **8**, incubation was performed at 21 °C.

Although it was clear that **8** acts as a targeted covalent inhibitor of HSP72, we had yet to confirm that Cys17 was the nucleophilic residue responsible, as predicted (Figure 2). To determine which reactive residue was forming the adduct, the pre‐incubated HSP72‐NBD/**8** mixture was subjected to trypsin‐digest MS/MS. Owing to the limitations of the MS analysis, it was necessary to focus the second MS fragmentation only on the three cysteine residues (Figure 6 and the Supporting Information). The MS1 and MS2 data unexpectedly revealed no evidence of Cys17 modification. However, a mass consistent with modification of the peptide TAC^267^ER was observed and the MS2 spectrum confirmed the modification present on buried Cys267, the residue proposed to be the target of YK5 **2** and oridonin **3**.

**Figure 6:**
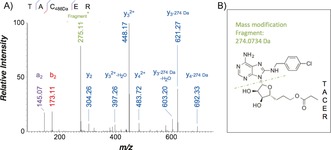
a) Trypsin‐digest MS/MS indicating modification of HSP72‐NBD with **8** at residue Cys267 (precursor ion 356.4756^3+^, error −0.84 ppm) b) Proposed schematic of fragmentation of **8** upon MS/MS.

The trypsin‐digest MS/MS data appeared to contradict our conclusion that the targeted covalent inhibitor **8** was actually acting in a specific manner, as it was unclear how the compound could react with Cys267 without a significant conformational change that should greatly disrupt the small‐molecule binding site. Therefore, we generated a cysteine‐to‐alanine mutant (C267A) of HSP72‐NBD, which bound **8** with a similar initial affinity to the wild‐type (FP‐assay, *K*_i_=16 μm, see the Supporting Information). However, when this mixture was analyzed by intact‐protein MS, **8** still formed a covalent adduct, eliminating Cys267 as the key nucleophilic residue. The C306A and C17A HSP72‐NBD mutants displayed similar results (see the Supporting Information).

We speculated that because the trypsin‐digest MS/MS data was not quantified, our original analysis had identified a minor adduct and that the modification of Cys267 only occurs readily once the protein denatures.^13^ As it was not possible to analyze all the nucleophilic residues with trypsin‐digest MS/MS, we used X‐ray crystallography in an attempt to predict which nucleophilic residues were sufficiently proximal to react with the electrophile, while conserving the 8‐*N*‐benzyladenosine binding mode (Figure 7).^17^

**Figure 7:**
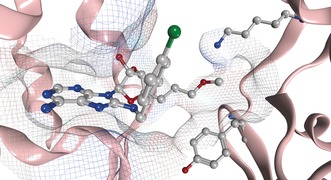
Targeted covalent inhibitor **8** reversibly bound to HSP72‐NBD at pH 8.8 (PDB: 5MKS, 2.0 Å), only key residues are shown, solvent and hydrogens omitted for clarity, carbon=grey, oxygen=red, nitrogen=blue, chlorine=green.

Despite significant efforts, no structure could be solved that describes the covalent complex. However, two co‐crystal structures were solved, which represent two different reversible binding modes of the ligand in the open conformation of HSP72‐NBD.^9^ In both modes, **8** demonstrated the expected hydrogen‐bonding array of the adenine and ribose moieties, with the lipophilic *para*‐chlorobenzylamine moiety parallel with the two α‐helices of the binding cleft.^4^ Interestingly, the acrylate electrophile was observed in two conformations, dependent on the crystallization conditions. At pH 4.1, the moiety points towards the front of the pocket, with the flexible Tyr15 in an up‐conformation (PDB: 5MKR, see the Supporting Information). At pH 8.8, Tyr15 is in a down‐conformation with the acrylate moiety now visible on a vector parallel with the base of the binding cleft (PDB: 5MKS, Figure 7 and the Supporting Information). The terminal portion of the acrylate itself could not be detected in the electron density, reflecting a high mobility. The closest potentially nucleophilic residue to the electrophilic warhead described in this structure is Lys56 (3.8 Å). To determine if Lys56 was the nucleophilic residue responsible for the irreversible inhibition,^18^ we repeated the trypsin‐digest MS/MS assay and found an MS1 mass consistent with modification of 22mer peptide Leu50–Lys71 (Figure 8). Unfortunately, no reliable MS2 spectrum could be acquired to confirm the site of modification within this peptide. Therefore, a K56A HSP72‐NBD mutant was used to confirm Lys56 as the key nucleophilic residue. This mutant displayed comparable apparent initial affinity for **8** (*K*_i_=11 μm)^18^ but in contrast to wild‐type HSP72‐NBD and the cysteine mutants, time‐dependence (24 h) was no longer observed in the FP‐assay and no significant modification was observed in the intact‐protein MS (Figure 8).

**Figure 8:**
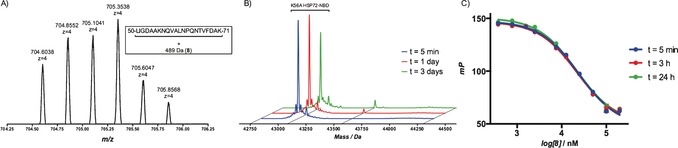
a) Extracted ion chromatogram of the MS1 from the trypsin digest of K56A HSP72‐NBD with **8**. Mass consistent with **8**‐modified 22mer, Leu50–Lys71, *z*=charge‐state. b) Intact‐protein MS trace of K56A HSP72‐NBD with **8** showing no significant time‐dependent modification. c) FP assay of K56A HSP72‐NBD with **8** showing no time‐dependent displacement of the fluorescent nucleotide probe.

This study began as the rational design of an irreversible inhibitor of HSP72 that would target Cys17 but ended with the identification of Lys56 as the key reacting nucleophilic residue. The involvement of non‐catalytic lysine residues as nucleophiles in covalent bond formation with targeted covalent inhibitors is rare.^19^ The discovery that Lys56, which is involved in a crucial salt‐bridge in HSP72,^9^ can undergo specific covalent bond formation with a validated inhibitor, opens up a new approach for antagonizing this challenging but important protein. We are currently exploring the potential of this new strategy through the design and synthesis of inhibitors that possess improved reversible affinity for HSP72 and electrophiles that are better matched to the lysine nucleophile.^19^ Once we have inhibitors with an acceptable profile, they will be tested in cellular assays to increase our understanding of the role of HSP72 in oncology.

## Conflict of interest

The authors declare no conflict of interest.

## Supporting information

As a service to our authors and readers, this journal provides supporting information supplied by the authors. Such materials are peer reviewed and may be re‐organized for online delivery, but are not copy‐edited or typeset. Technical support issues arising from supporting information (other than missing files) should be addressed to the authors.

Supplementary
