# Supplementary material for: An Irreversible Inhibitor of HSP72 that Unexpectedly Targets Lysine‐56
Source: Angew Chem Int Ed Engl. 2017 Feb 22;56(13):3536–40. doi: 10.1002/anie.201611907 (PMC5412842; doi:10.1002/anie.201611907)
Supplement: Supplementary file 1 — Supplementary [file ANIE-56-3536-s001.pdf]

Angewandte Chemie
Eine Zeitschrift der Gesellschaft Deutscher Chemiker

Supporting Information

An Irreversible Inhibitor of HSP72 that Unexpectedly Targets Lysine-56

*Jonathan Pettinger, Yann-Vaï Le Bihan, Marcella Widya, Rob L. M. van Montfort, Keith Jones, and Matthew D. Cheeseman**

anie_201611907_sm_miscellaneous_information.pdf

Author Contributions

J.P. Conceptualization: Equal; Formal analysis: Equal; Investigation: Lead; Methodology: Lead; Validation: Equal; Visualization: Equal; Writing—original draft: Supporting; Writing—review & editing: Equal
Y.L. Formal analysis: Supporting; Supervision: Supporting; Validation: Equal; Writing—review & editing: Equal
M.W. Formal analysis: Equal; Investigation: Equal; Methodology: Equal; Validation: Supporting; Visualization: Supporting; Writing—review & editing: Equal
R.v. Formal analysis: Supporting; Funding acquisition: Equal; Project administration: Supporting; Resources: Equal; Supervision: Supporting; Validation: Supporting; Writing—review & editing: Supporting
K.J. Conceptualization: Equal; Formal analysis: Supporting; Funding acquisition: Lead; Methodology: Supporting; Project administration: Supporting; Resources: Lead; Supervision: Equal; Validation: Supporting; Writing—review & editing: Equal
M.C. Conceptualization: Lead; Formal analysis: Lead; Methodology: Lead; Project administration: Supporting; Supervision: Lead; Validation: Equal; Visualization: Lead; Writing—original draft: Lead; Writing—review & editing: Lead.

Supporting Information

Table of Contents

General Chemistry Experimental	S2
Chemistry Experimental Procedures	S3 – S11
NMR Spectra	S12 – S22
Protein Production and Purification Experimental	S23 – S24
Fluorescence Polarization Assay Experimental	S25 – S26
Fluorescence Polarization Assay Data	S27 – S34
HSP72 Protein Mass Spectrometry Experimental	S35 – S36
HSP72 Intact-Protein Mass Spectrometry Data	S37 – S40
HSP72 Trypsin-Digest Mass Spectrometry Data	S41 – S42
HSP70 Protein Crystal Structure Analysis	S43
HSP72-NBD Protein Crystallography Experimental	S44
Crystallographic Data Collection and Refinement Statistics	S45
HSP72 Protein Crystallography Images	S46 – S48
Structure of HSP72-SBD Covalent Inhibitor Novolactone	S49
Structure of HSP72-NBD Reversible Inhibitor Ver-155008	S49
Full Authorship List From Paper References	S50
References	S51

General Chemistry Experimental

Unless otherwise stated, all reactions were conducted in oven-dried glassware under an inert atmosphere of nitrogen using anhydrous solvents. All commercially obtained reagents and solvents were used as received. Oridonin **3** was purchased from Sigma-Aldrich. Microwave reactions were performed using a Biotage Initiator Microwave Synthesizer. Thin-layer chromatography (TLC) was performed on precoated aluminium sheets of silica (60 F254 nm, Merck) and visualized using short-wave UV light. Column chromatography was performed on a Biotage SP1 purification system using Biotage Flash silica cartridges. All melting points were determined on a Stanford Research Systems EZ-melt apparatus and are uncorrected. Optical rotations were recorded on a Bellingham & Stanley Ltd. ADP440 Polarimeter with a path length of 0.1 dm, using a light emitting diode with interference filter (298 nm). Concentrations (*c*) are quoted in g/100 mL. ^{1}H NMR spectra were recorded on Bruker AMX500 (500 MHz) spectrometers using an internal deuterium lock. Chemical shifts are quoted in parts per million (ppm) using the following internal references: $CDCl_3$ (δH 7.26), MeOD (δH 3.31) and DMSO-d_6 (δH 2.50). Signal multiplicities are recorded as singlet (s), doublet (d), triplet (t), quartet (q), multiplet (m), doublet of doublets (dd), doublet of doublet of doublets (ddd), triplet of doublets (td), apparent (app.), broad (br.) or obscured (obs.). Coupling constants, *J*, are measured to the nearest 0.1 Hz. ^{13}C NMR spectra were recorded on Bruker AMX500 spectrometers at 126 MHz using an internal deuterium lock. Chemical shifts are quoted to 0.01 ppm, using the following internal references: $CDCl_3$ (δC 77.0), MeOD (δC 49.0) and DMSO-d_6 (δC 39.5). High-resolution mass spectra were recorded on an Agilent 1200 series HPLC and diode array detector coupled to a 6210 time-of-flight mass spectrometer with dual multimode APCI/ESI source. Analytical separation was carried out on a Merck Purospher STAR RP-18, 30 mm x 4 mm column using a flow rate of 1.5 mL/min in a 4 min gradient elution, UV detection was at 254 nm. Infrared spectra were recorded on a Bruker Alpha-p FT-IR spectrometer. Only structurally important absorption peaks are quoted, absorption maxima (V_{max}) are quoted in wavenumbers (cm^{-1}). All compounds were >95% purity by HPLC analysis unless otherwise stated.

Chemistry Experimental Procedures

(2*R*,3*R*,4*S*,5*R*)-2-(6-Amino-8-bromo-9*H*-purin-9-yl)-5-(hydroxymethyl) tetrahydrofuran-3,4-diol (S1)

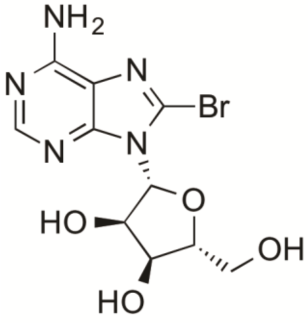

S1

Adenosine (659 mg, 2.47 mmol) was dissolved in dioxane (12 mL) and water (12 mL). $K_2HPO_4.3H_2O$ (1.69 g, 7.40 mmol) was dissolved in water (12 mL) then added to bromine (0.990 g, 6.16 mmol). The bromine solution was added dropwise to the stirred adenosine solution. After 15 minutes, the reaction was quenched with sat. $Na_2S_2O_3$ solution (10 mL) and stirred for a further 2 minutes. The resulting mixture was extracted with EtOAc (3 x 50 mL), then the combined organic layers washed with sat. brine (3 x 50 mL) and dried over $MgSO_4$. The solvent was removed under reduced pressure to give the title compound **S1** as a white solid which was carried on to the next step without further purification (370 mg, 41%); **mp** decomposed 210 °C; **$[\alpha]^{22}_D$** = -16.6 ° (*c* 0.5, DMSO); **^{1}H NMR** (500 MHz, DMSO-d^6) δH 8.12 (s, 1H), 7.59 (br. s, 2H), 5.83 (d, *J* = 6.8 Hz, 1H), 5.52 (br. s, 1H), 5.47 (d, *J* = 6.0 Hz, 1H), 5.25 (br. s, 1H), 5.09 (m, 1H), 4.19 (dd, *J* = 5.2, 2.3 Hz, 1H), 3.98 (app. td, *J* = 4.0, 2.3 Hz, 1H), 3.68 (dd, *J* = 12.1, 4.0 Hz, 1H), 3.52 (d, *J* = 12.3 Hz, 1H); **^{13}C NMR** (126 MHz, DMSO) δC 155.15, 152.42, 149.84, 127.21, 119.67, 90.38, 86.73, 71.11, 70.89, 62.12; **HRMS** (ESI) $C_{10}H_{13}N_5O_4{}^{79}Br$ ($M+H^+$) requires 346.0145, found 346.0137; ***t_R*** (LCMS) = 0.42 min; **IR** (FTIR-ATR)/cm^{-1} = 3484, 3378, 3212, 2923, 1642, 1579.

(2*R*,3*R*,4*S*,5*R*)-2-(6-Amino-8-((4-chlorobenzyl)amino)-9*H*-purin-9-yl)-5-(hydroxymethyl)tetrahydrofuran-3,4-diol (1)

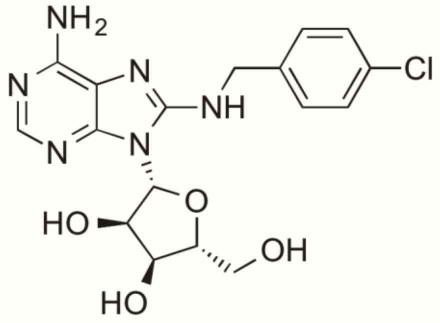

1

8-Bromoadenosine **S1** (75 mg, 0.220 mmol) and 4-chlorobenzylamine (153 mg, 1.08 mmol) were dissolved in EtOH (2.2 mL). The reaction was heated in the microwave for 1 hour at 160 °C. The solvent was removed under reduced pressure and the resulting residue taken up in EtOAc (20 mL) and water (20 mL). The product was extracted with EtOAc (3 x 20 mL) then washed with sat. brine (3 x 20 mL) and dried over $MgSO_4$. The solvent was removed under reduced pressure to give an orange solid. The crude product was purified by silica gel chromatography with the Biotage SP1 purification system (EtOAc/EtOH 95:5 to 80:20) to give the title compound **1** as an orange solid (49 mg, 56%); $[\alpha]^{20}_D$ = -15.9 (*c* 1, MeOH); ^{1}**H NMR** (500 MHz, MeOD) δH 7.97 (s, 1H), 7.37 (d, *J* = 8.6 Hz, 2H), 7.32 (d, *J* = 8.5 Hz, 2H), 6.08 (d, *J* = 7.6 Hz, 1H), 4.77 (dd, *J* = 7.6, 5.4 Hz, 1H), 4.65 – 4.57 (m, 2H), 4.28 (dd, *J* = 5.4, 1.7 Hz, 1H), 4.16 (app. q, *J* = 1.9 Hz, 1H), 3.84 – 3.75 (m, 2H); ^{13}**C NMR** (126 MHz, MeOD) δC 153.59, 153.22, 151.08, 150.12, 139.06, 133.82, 129.63, 129.56, 117.95, 88.71, 87.80, 73.06, 72.90, 63.13, 46.21; **HRMS** (ESI) $C_{17}H_{20}N_6O_4{}^{35}Cl$ $(M+H^+)$ requires 407.1229, found 407.1222; t_R (LCMS) = 1.13 min; **IR** (FTIR-ATR)/cm^{-1} = 3311, 2926, 1641, 1614, 1575.

Ethyl(*E*)-3-((3a*R*,4*R*,6*R*,6a*R*)-6-(6-amino-9*H*-purin-9-yl)-2,2-dimethyltetrahydrofuro [3,4-*d*][1,3]dioxol-4-yl)acrylate (S2)

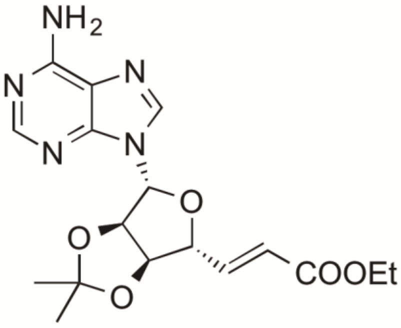

S2

2′,3′-*O*-Isopropylideneadenosine (1.97 g, 6.41 mmol) was dissolved in DMSO (16 mL). 45 wt% IBX (10.0 g, 16.0 mmol) and (carbethoxymethylene) triphenylphosphorane (5.58 g, 16.0 mmol) were added giving a white suspension. The reaction was stirred in the dark for 73 hours. Water (70 mL) was added and the resulting suspension extracted with EtOAc (5 x 50 mL). The combined organic layers were washed with sat. brine (5 x 50 mL) and dried over Na_2SO_4. The solvent was removed under reduced pressure and the crude product purified by silica gel chromatography with the Biotage SP1 purification system (EtOAc/EtOH 95:5 to 90:10) to give the title compound **S2** as an orange solid (1.50 g, 63%); **mp** 73-75 °C; $[\alpha]^{22}_D$ = +23.5 ° (*c* 0.5, $CHCl_3$); ^{1}**H NMR** (500 MHz, $CDCl_3$) δH 8.32 (s, 1H), 7.87 (s, 1H), 6.96 (dd, *J* = 15.7, 5.6 Hz, 1H), 6.13 (d, *J* = 1.9 Hz, 1H), 5.94 (br. s, 2H), 5.81 (dd, *J* = 15.7, 1.7 Hz, 1H), 5.56 (dd, *J* = 6.3, 1.9 Hz, 1H), 5.14 (dd, *J* = 6.2, 3.6 Hz, 1H), 4.81 (ddd, *J* = 5.4, 3.6, 1.7 Hz,

1H), 4.11 (app. q, *J* = 7.2 Hz, 2H), 1.62 (s, 3H), 1.40 (s, 3H), 1.22 (app. t, *J* = 7.2 Hz, 3H); **^{13}C NMR** (126 MHz, $CDCl_3$) δC 165.70, 155.75, 153.28, 149.53, 143.53, 140.09, 122.69, 120.37, 114.83, 90.71, 86.40, 84.47, 84.07, 60.70, 27.22, 25.48, 14.27.; **HRMS** (ESI) $C_{17}H_{22}N_5O_5$ (M+H$^+$) requires 376.1615, found 376.1614; ***t*$_R$** (LCMS) = 1.27 min; **IR** (FTIR-ATR)/cm^{-1} = 3326, 3174, 2986, 1716, 1648, 1599.

(*E*)-3-((3a*R*,4*R*,6*R*,6a*R*)-6-(6-Amino-9*H*-purin-9-yl)-2,2-dimethyltetrahydrofuro[3,4-*d*][1,3]dioxol-4-yl)prop-2-en-1-ol (S3)

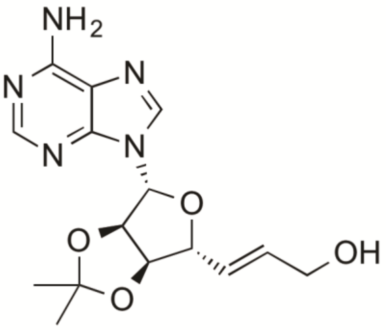

S3

Unsaturated ester **S2** (2.03 g, 5.42 mmol) was dissolved in dry CH_2Cl_2 (18 mL) at -78 °C. DIBAL-H (1 M in toluene, 43.3 mL, 43.3 mmol) was added dropwise to the solution at -78 °C and stirred for 2 hours, then quenched with MeOH (20 mL) and warmed to room temperature. A sat. solution of Rochelle's Salt (150 mL) was added and the resulting suspension stirred overnight. The mixture was extracted with EtOAc (3 x 50 mL) and the combined organic layers were washed with sat. brine (3 x 50 mL) and dried over Na_2SO_4. The solvent was removed under reduced pressure to give the title compound **S3** as a white foam, which was used without further purification (1.53 g); **$[\alpha]^{21}_D$** = +33.2 ° (*c* 0.1, $CHCl_3$); **^{1}H NMR** (500 MHz, $CDCl_3$) δH 8.30 (s, 1H), 8.01 (s, 1H), 6.97 (br. s, 2H), 6.12 (d, *J* = 1.9 Hz, 1H), 5.92 – 5.85 (m, 2H), 5.49 (dd, *J* = 6.2, 1.9 Hz, 1H), 5.00 (dd, *J* = 6.2, 2.8 Hz, 1H), 4.80 (m, 1H), 4.22 (br. s, 1H), 4.14 – 4.08 (obs. m, 2H), 1.62 (s, 3H), 1.39 (s, 3H); **^{13}C NMR** (126 MHz, $CDCl_3$) δC 155.56, 153.23, 149.51, 140.09, 134.15, 127.38, 120.23, 114.58, 90.79, 87.82, 84.77, 84.39, 62.30, 27.24, 25.51; **HRMS** (ESI) $C_{15}H_{20}N_5O_4$ (M+H$^+$) requires 334.1510, found 334.1504; ***t*$_R$** (LCMS) = 0.96 min; **IR** (FTIR-ATR)/cm^{-1} = 3335, 3188, 2988, 2918, 1644, 1599.

3-((3a*R*,4*R*,6*R*,6a*R*)-6-(6-Amino-9*H*-purin-9-yl)-2,2-dimethyltetrahydrofuro[3,4-*d*][1,3]dioxol-4-yl)propan-1-ol (5)

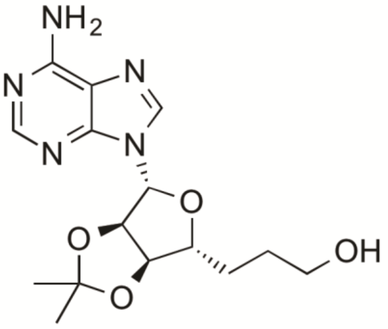

5

A solution of allyl alcohol **S3** (1.54 g, 4.62 mmol) in EtOH (46 mL) was added to 10% Pd/C (0.250 g) and stirred vigorously to generate a deep vortex in a hydrogen atmosphere (5.2 atm) at room temperature for 20 hours. The catalyst was removed by filtration over Celite and washed with EtOH (10 mL). The solvent was removed under reduced pressure to give a white foam, which was purified by silica gel chromatography with the Biotage SP1 purification system (EtOAc/EtOH 95:5 to 85:15) to give the title compound **5** as a white foam (0.650 g, 85% over two steps); **mp** 178 – 182 °C; $[\alpha]^{22}_{D}$ = +11.1 ° (*c* 0.5, $CHCl_3$); **^{1}H NMR** (500 MHz, $CDCl_3$) δH 8.32 (s, 1H), 7.88 (s, 1H), 6.05 (br. obs. s, 2H), 6.03 (obs. d, *J* = 2.4 Hz, 1H), 5.48 (dd, *J* = 6.5, 2.4 Hz, 1H), 4.84 (dd, *J* = 6.5, 3.7 Hz, 1H), 4.22 (app. td, *J* = 7.1, 3.8 Hz, 1H), 3.60 (app. td, *J* = 6.3, 1.2 Hz, 2H), 2.53 (br. s, 1H), 1.81 – 1.75 (m, 2H), 1.68 – 1.61 (obs. m, 2H), 1.60 (obs. s, 3H), 1.38 (s, 3H); **^{13}C NMR** (126 MHz, $CDCl_3$) δC 155.75, 153.31, 149.49, 139.80, 120.32, 114.76, 90.51, 86.94, 84.31, 84.14, 62.17, 30.08, 28.93, 27.31, 25.55; **HRMS** (ESI) $C_{15}H_{22}N_5O_4$ (M+H^+) requires 336.1666, found 336.1661; t_R (LCMS) = 1.01 min; **IR** (FTIR-ATR)/cm^{-1} = 3329, 3179, 2988, 2937, 1645, 1599.

3-((3a*R*,4*R*,6*R*,6a*R*)-6-(6-Amino-8-bromo-9*H*-purin-9-yl)-2,2-dimethyltetrahydrofuro [3,4-*d*][1,3]dioxol-4-yl)propan-1-ol (6)

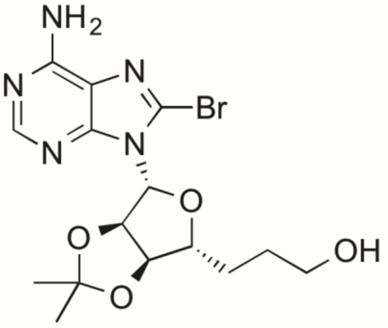

6

Primary alcohol **5** (0.560 g, 1.68 mmol) was dissolved in dioxane (17 mL). A solution of $K_2HPO_4.3H_2O$ (1.15 g, 5.05 mmol) and bromine (0.670 g, 4.20 mmol) in water (17 mL) was then added dropwise to the stirred solution of **5**. After 15 minutes, the reaction was quenched with sat. $Na_2S_2O_3$ solution (5 mL) and stirred for a further 2 minutes. The resulting mixture was extracted with EtOAc (3 x 30 mL), then the combined organic layers were washed with sat. brine (3 x 30 mL) and dried over $MgSO_4$. The solvent was removed under reduced pressure to give the title compound **6** as an orange foam which was used in the next step without further purification (0.670 g, 91%); **$[\alpha]^{22}_D$** = -8.3 ° (*c* 0.5, $CHCl_3$); **^{1}H NMR** (500 MHz, $CDCl_3$) δH 8.32 (s, 1H), 6.87 (br. s, 2H), 6.15 (d, *J* = 2.2 Hz, 1H), 5.68 (dd, *J* = 6.4, 2.2 Hz, 1H), 4.96 (dd, *J* = 6.4, 4.0 Hz, 1H), 4.20 (ddd, *J* = 7.9, 6.4, 4.0 Hz, 1H), 3.58 (app. t, *J* = 6.2 Hz, 2H), 1.79 – 1.71 (m, 2H), 1.64 – 1.57 (obs. m, 2H), 1.61 (obs. s, 3H), 1.39 (s, 3H); **^{13}C NMR** (126 MHz, $CDCl_3$) δ 154.33, 153.10, 150.75, 127.65, 120.31, 114.54, 90.83, 87.52, 84.50, 83.38, 62.46, 29.78, 29.00, 27.39, 25.61; **HRMS** (ESI) $C_{15}H_{21}N_5O_4{}^{79}Br$ (M+H$^+$) requires 414.0771, found 414.0760; ***t_R*** (LCMS) = 1.28 min; **IR** (FTIR-ATR)/cm^{-1} = 3330, 3184, 2987, 2939, 1647, 1601.

3-((3a*R*,4*R*,6*R*,6a*R*)-6-(6-Amino-8-((4-chlorobenzyl)amino)-9*H*-purin-9-yl)-2,2-dimethyltetrahydrofuro[3,4-*d*][1,3]dioxol-4-yl)propan-1-ol (7)

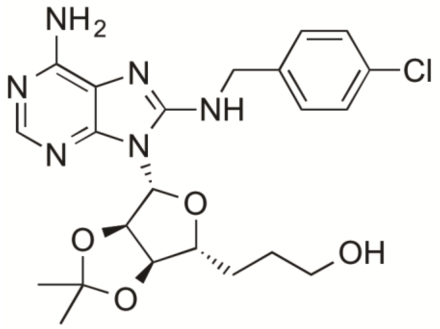

7

8-Bromoadenosine derivative **6** (96 mg, 0.230 mmol) was dissolved in EtOH (2.3 mL) and added to 4-chlorobenzylamine (0.330 g, 2.32 mmol) and the reaction was heated in the microwave for 30 minutes at 170 °C. The solvent was removed under reduced pressure and the resulting residue taken up in CH_2Cl_2 (10 mL) and water (10 mL). The product was extracted with CH_2Cl_2 (3 x 10 mL), washed with sat. brine (3 x 10 mL) and dried over Na_2SO_4. The solvent was removed under reduced pressure and the crude product was purified by silica gel chromatography with the Biotage SP1 purification system (EtOAc/EtOH 100:0 to 80:20) to give the title compound **7** as an orange solid (0.110 g, 98%); **mp** 91 – 94 °C; **$[\alpha]^{22}_D$** = -87.3 ° (*c* 0.5, $CHCl_3$); **^{1}H NMR** (500 MHz, $CDCl_3$) δH 8.16 (s, 1H), 7.31 (app. s, 4H), 5.95 (dd, *J* = 6.4, 2.3 Hz, 1H), 5.90 (d, *J* = 2.2 Hz, 1H), 5.29 (br. s, 2H), 5.20 (t, *J* = 5.7

Hz, 1H), 4.74 (dd, *J* = 6.4, 3.3 Hz, 1H), 4.58 (dd, *J* = 5.6, 3.7 Hz, 2H), 4.15 (m, 1H), 3.50 – 3.46 (m, 2H), 1.94 (br. s, 2H), 1.56 (obs. s, 3H), 1.59 – 1.44 (obs. m, 4H), 1.38 (s, 3H); **^{13}C NMR** (126 MHz, $CDCl_3$) δC 152.38, 151.88, 150.21, 150.05, 136.78, 133.69, 129.33, 129.04, 117.65, 114.42, 89.26, 86.85, 84.34, 82.27, 62.04, 46.52, 29.75, 28.82, 27.12, 25.42; **HRMS** (ESI) $C_{22}H_{28}N_6O_4{}^{35}Cl$ (M+H$^+$) requires 475.1855, found 475.1845; t_R (LCMS) = 1.41 min; **IR** (FTIR-ATR)/cm^{-1} = 3337, 2935, 1638, 1608, 1573.

3-((3a*R*,4*R*,6*R*,6a*R*)-6-(6-Amino-8-((4-chlorobenzyl)amino)-9*H*-purin-9-yl)-2,2-dimethyltetrahydrofuro[3,4-*d*][1,3]dioxol-4-yl)propyl acrylate (10)

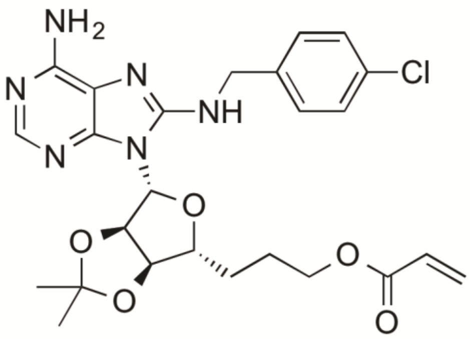

10

Primary alcohol **7** (102 mg, 0.210 mmol) was dissolved in a solution of triethylamine (24 mg, 0.240 mmol) and CH_2Cl_2 (2 mL) and cooled to 0 °C. Acryloyl chloride (22 mg, 0.240 mmol) in CH_2Cl_2 (1 mL) was added dropwise to the solution and stirred for 6 hours, then quenched with sat. $NaHCO_3$ (1.5 mL). The solvent was removed under reduced pressure and the subsequent residue taken up in EtOAc (10 mL), washed with sat. brine (3 x 10 mL) and dried over $MgSO_4$. The solvent was removed under reduced pressure to give the crude product, which was purified by silica gel chromatography with the Biotage SP1 purification system (EtOAc/EtOH 100:0 to 90:10) to give the title compound **10** as a colourless oil (17 mg, 14%); **$[\alpha]^{22}_D$** = -22.2 ° (*c* 0.5, $CHCl_3$); **^{1}H NMR** (500 MHz, $CDCl_3$) δH 8.15 (s, 1H), 7.32 (app. s, 4H), 6.30 (dd, *J* = 17.3, 1.4 Hz, 1H), 6.04 (m, 1H), 6.00 (dd, *J* = 17.3, 10.4 Hz, 1H), 5.87 (d, *J* = 2.0 Hz, 1H), 5.79 (dd, *J* = 10.5, 1.5 Hz, 1H), 5.29 (br. s, 2H), 5.12 (t, *J* = 5.7 Hz, 1H), 4.78 (dd, *J* = 6.4, 3.1 Hz, 1H), 4.61 (d, *J* = 5.6 Hz, 2H), 4.15 (ddd, *J* = 8.6, 5.7, 3.1 Hz, 1H), 4.06 – 3.97 (m, 2H), 1.65 – 1.58 (obs. m, 2H), 1.56 (obs. s, 3H), 1.55 – 1.44 (obs. m, 2H), 1.38 (s, 3H); **^{13}C NMR** (126 MHz, $CDCl_3$) δC 166.19, 152.40, 151.89, 150.11, 149.99, 136.81, 133.70, 130.95, 129.31, 129.02, 128.34, 117.75, 114.28, 89.27, 86.93, 84.41, 82.27, 63.77, 46.53, 29.83, 27.08, 25.41, 25.13; **HRMS** (ESI) $C_{25}H_{30}N_6O_5{}^{35}Cl$ (M+H$^+$) requires 529.1966, found 529.1976; t_R (LCMS) = 1.54 min; **IR** (FTIR-ATR)/cm^{-1} = 3341, 2934, 1721, 1637, 1609, 1573.

3-((2*R*,3*S*,4*R*,5*R*)-5-(6-Amino-8-((4-chlorobenzyl)amino)-9*H*-purin-9-yl)-3,4-dihydroxytetrahydrofuran-2-yl)propyl acrylate (8)

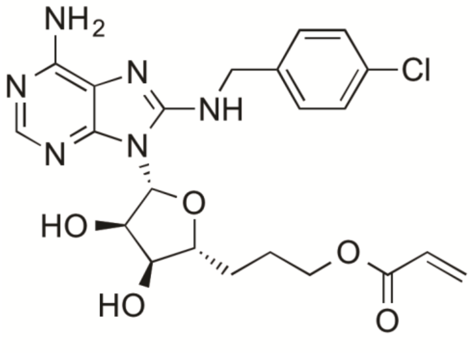

8

Acrylate **10** (20 mg, 0.040 mmol) was dissolved in a 5:2 mixture of TFA/H_2O (1.4 mL) and stirred at room temperature for 30 minutes. The solvent was removed under reduced pressure to give the crude product, which was purified by silica gel chromatography with the Biotage SP1 purification system (EtOAc/EtOH 95:5 to 80:20) to give the title compound **8** as a colourless oil (18 mg, 91%); $[\alpha]^{22}_D$ = +83.1 ° (*c* 0.5, MeOH); **^{1}H NMR** (500 MHz, MeOD) δH 7.98 (s, 1H), 7.38 (d, *J* = 8.6 Hz, 2H), 7.33 (d, *J* = 8.5 Hz, 2H), 6.33 (dd, *J* = 17.3, 1.4 Hz, 1H), 6.10 (dd, *J* = 17.3, 10.4 Hz, 1H), 5.84 (dd, *J* = 10.4, 1.6 Hz, 1H), 5.78 (d, *J* = 4.9 Hz, 1H), 5.24 (dd, *J* = 5.9, 4.8 Hz, 1H), 4.63 (app. s, 2H), 4.28 (app. t, *J* = 5.5 Hz, 1H), 4.16 – 4.11 (m, 2H), 3.95 (m, 1H), 1.84 – 1.72 (m, 4H); **^{13}C NMR** (126 MHz, MeOD) δC 167.74, 153.89, 153.56, 151.19, 150.36, 138.97, 133.94, 131.38, 129.73, 129.61, 129.55, 118.17, 89.56, 84.93, 74.79, 72.53, 65.48, 46.43, 30.73, 26.20; **HRMS** (ESI) $C_{22}H_{26}N_6O_5{}^{35}Cl$ (M+H$^+$) requires 489.1648, found 489.1642; t_R (LCMS) = 1.35 min; **IR** (FTIR-ATR)/cm^{-1} = 3341, 2926, 1717, 1638, 1612, 1573.

3-((3a*R*,4*R*,6*R*,6a*R*)-6-(6-Amino-8-((4-chlorobenzyl)amino)-9*H*-purin-9-yl)-2,2-dimethyltetrahydrofuro[3,4-*d*][1,3]dioxol-4-yl)propyl propionate (S4)

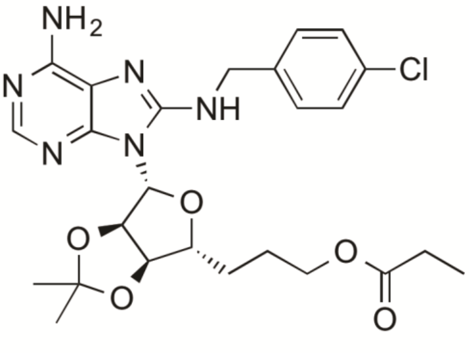

S4

Primary alcohol **7** (74 mg, 0.160 mmol) was dissolved in a solution of triethylamine (32 mg, 0.320 mmol) and CH_2Cl_2 (3.0 mL) and cooled to 0 °C. Propionyl chloride (0.03 mL, 0.320 mmol) was added dropwise to the solution and stirred for 30 minutes. The reaction was quenched with sat. $NaHCO_3$ (1 mL), and the solvent was removed under reduced pressure. The resulting residue was taken up in EtOAc (10 mL), washed with sat. brine (3 x 10 mL) and dried over $MgSO_4$. The solvent was removed under reduced pressure and the crude product was purified by silica gel chromatography with the Biotage SP1 purification system (EtOAc/EtOH 100:0 to 90:10) to give the title compound **S4** as a colourless oil (45 mg, 54%); **$[\alpha]^{21}_D$** = -5.5 ° (*c* 0.5, $CHCl_3$); **^{1}H NMR** (500 MHz, $CDCl_3$) δH 8.15 (s, 1H), 7.32 (app. s, 4H), 6.04 (dd, *J* = 6.4, 2.0 Hz, 1H), 5.87 (d, *J* = 2.0 Hz, 1H), 5.29 (app. s, 2H), 5.12 (t, *J* = 5.7 Hz, 1H), 4.77 (dd, *J* = 6.1, 3.1 Hz, 1H), 4.61 (dd, *J* = 5.6, 0.9 Hz, 2H), 4.14 (m, 1H), 3.96 – 3.90 (m, 2H), 2.21 (app. q, *J* = 7.6 Hz, 2H), 1.56 (obs. s, 3H), 1.61 – 1.41 (obs. m, 4H), 1.38 (s, 3H), 1.07 (app. t, *J* = 7.6 Hz, 3H); **^{13}C NMR** (126 MHz, $CDCl_3$) δC 174.52, 152.40, 151.90, 150.12, 150.00, 136.80, 133.71, 129.30, 129.03, 117.71, 114.27, 89.28, 86.96, 84.41, 82.26, 63.56, 46.53, 29.85, 27.55, 27.08, 25.41, 25.14, 9.21; **HRMS** (ESI) $C_{25}H_{32}N_6O_5{}^{35}Cl$ ($M+H^+$) requires 531.2117, found 531.2103; ***t_R*** (LCMS) = 1.55 min; **IR** (FTIR-ATR)/cm^{-1} = 3344, 2984, 1732, 1637, 1608, 1574.

3-((2*R*,3*S*,4*R*,5*R*)-5-(6-Amino-8-((4-chlorobenzyl)amino)-9*H*-purin-9-yl)-3,4-dihydroxytetrahydrofuran-2-yl)propyl propionate (9)

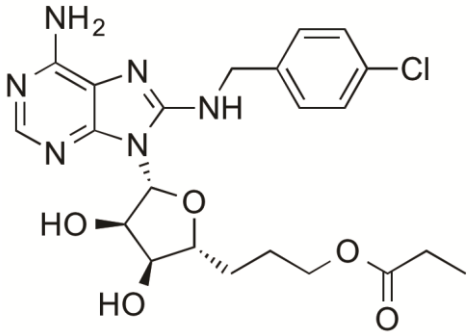

9

Propionyl ester **S4** (11 mg, 21 μmol) was dissolved in a 5:2 mixture of TFA/H_2O (1.4 mL) and stirred at room temperature for 30 minutes. The solvent was then removed under reduced pressure to give the crude product, which was purified by silica gel chromatography with the Biotage SP1 purification system (EtOAc/EtOH 95:5 to 80:20) to give the title compound **9** as a colourless oil (7 mg, 48%); **$[\alpha]^{21}_D$** = +8.3 ° (*c* 0.5, MeOH); **^{1}H NMR** (500 MHz, MeOD) δH 7.99 (s, 1H), 7.38 (d, *J* = 8.6 Hz, 2H), 7.33 (d, *J* = 8.5 Hz, 2H), 5.79 (d, *J* = 4.9 Hz, 1H), 5.23 (dd, *J* = 5.9, 4.9 Hz, 1H), 4.63 (app. s, 2H), 4.29 (app. t, *J* = 5.5 Hz, 1H), 4.07 – 4.02 (m, 2H), 3.95 (m, 1H), 2.28 (app. q, *J* = 7.5 Hz, 2H), 1.81 – 1.68 (m, 4H), 1.06

(app. t, J = 7.6 Hz, 3H); **^{13}C NMR** (126 MHz, MeOD) δC 176.23, 153.88, 153.51, 151.16, 150.28, 138.95, 133.94, 129.74, 129.60, 118.16, 89.53, 84.97, 74.77, 72.59, 65.26, 46.44, 30.72, 28.28, 26.19, 9.43; **HRMS** (ESI) $C_{22}H_{28}N_6O_5{}^{35}Cl$ (M+H$^+$) requires 491.1804, found 491.1792; ***t_R*** (LCMS) = 1.38 min; **IR** (FTIR-ATR)/cm^{-1} = 3336, 2924, 1723, 1640, 1613, 1574.

NMR Spectra

(2*R*,3*R*,4*S*,5*R*)-2-(6-Amino-8-bromo-9*H*-purin-9-yl)-5-(hydroxymethyl) tetrahydrofuran-3,4-diol (S1)

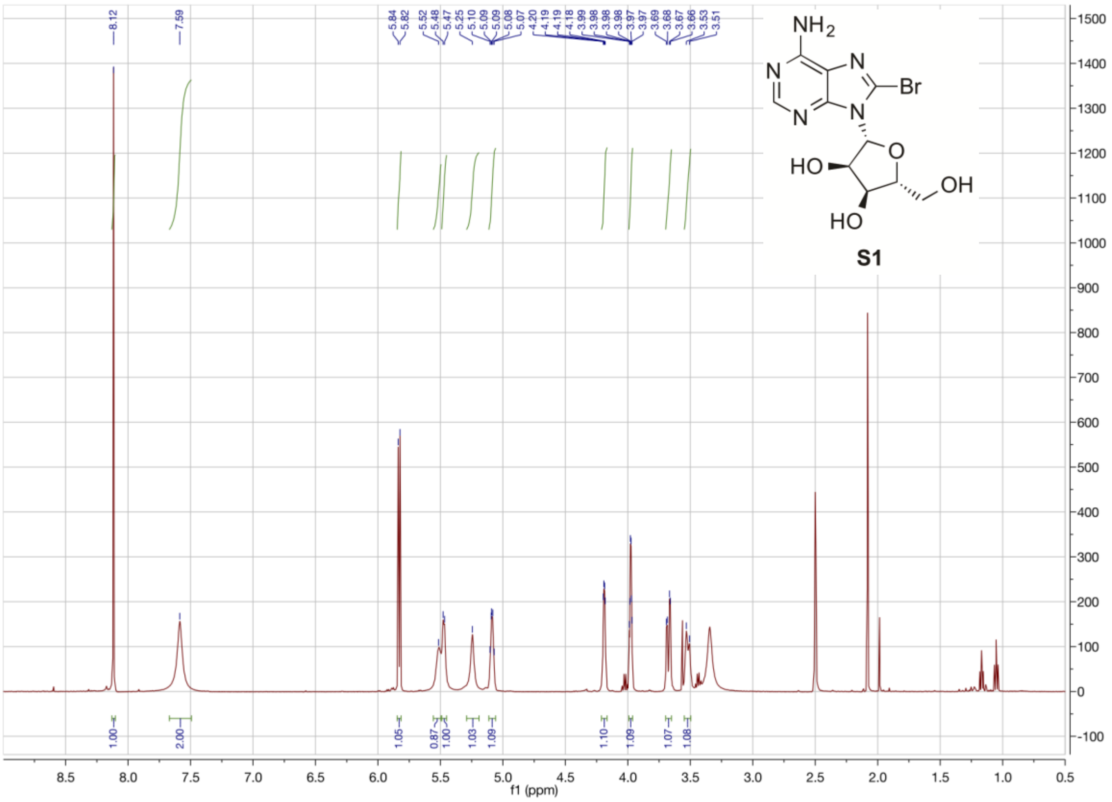

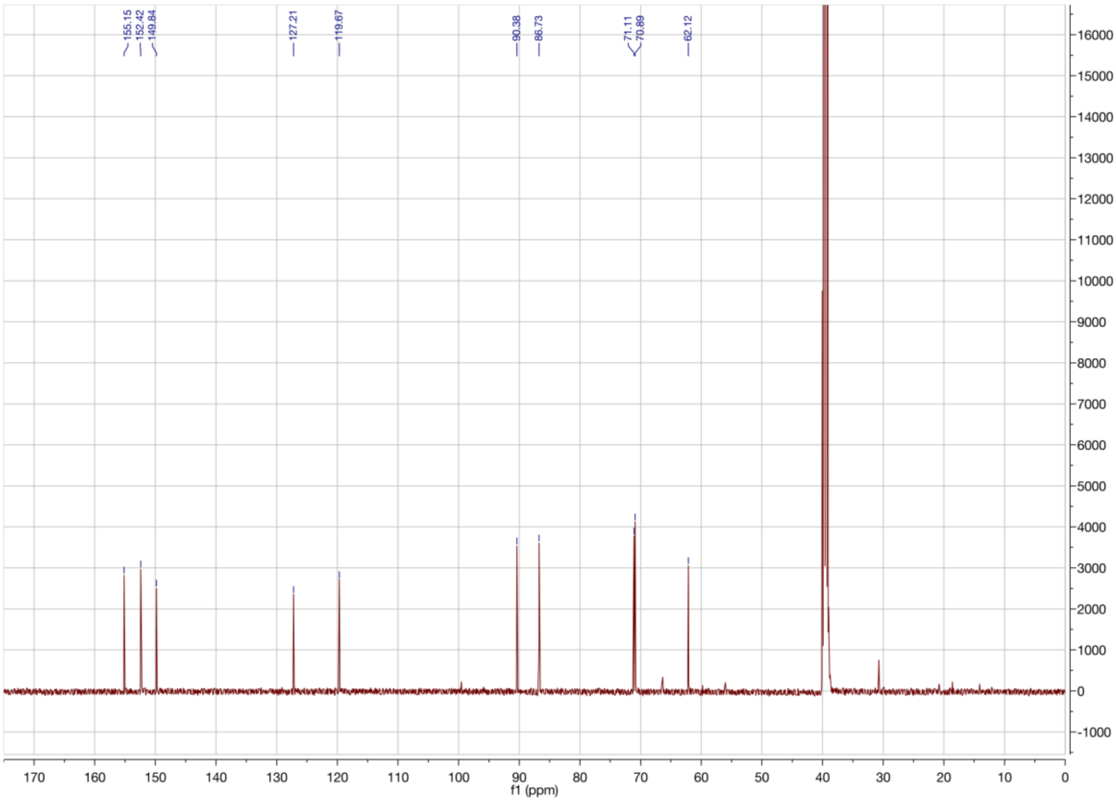

(2*R*,3*R*,4*S*,5*R*)-2-(6-Amino-8-((4-chlorobenzyl)amino)-9*H*-purin-9-yl)-5-(hydroxymethyl)tetrahydrofuran-3,4-diol (1)

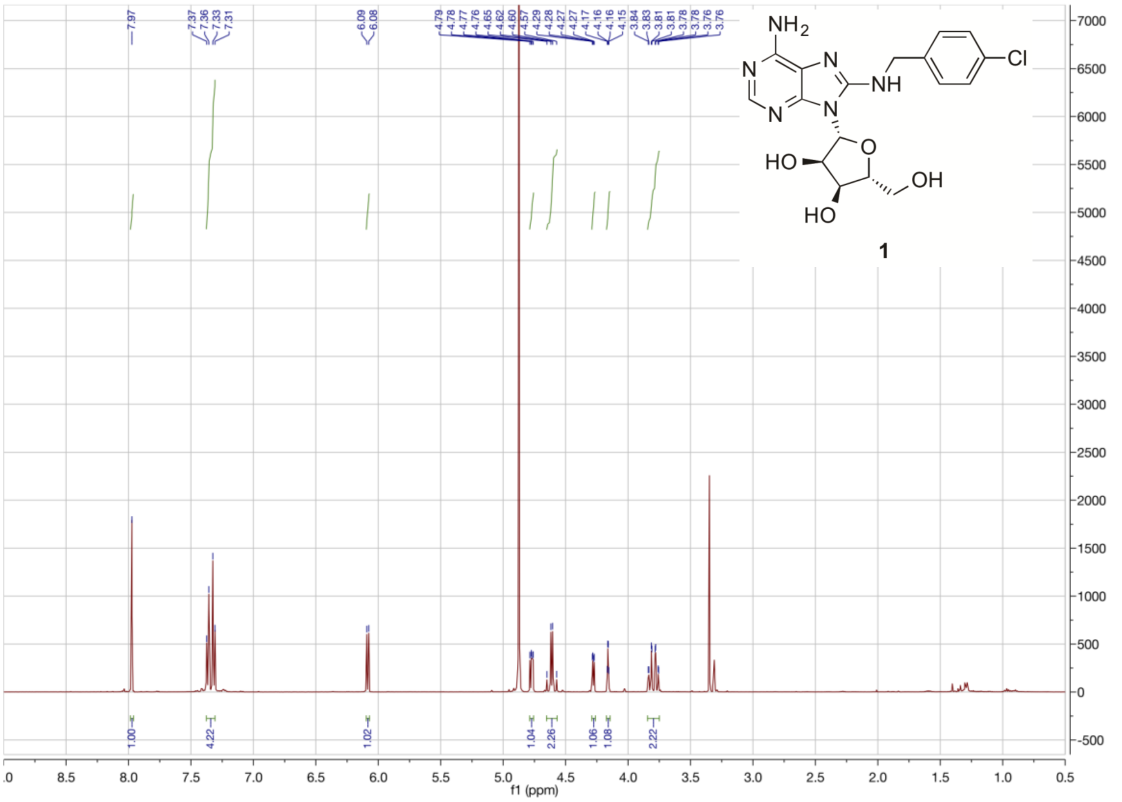

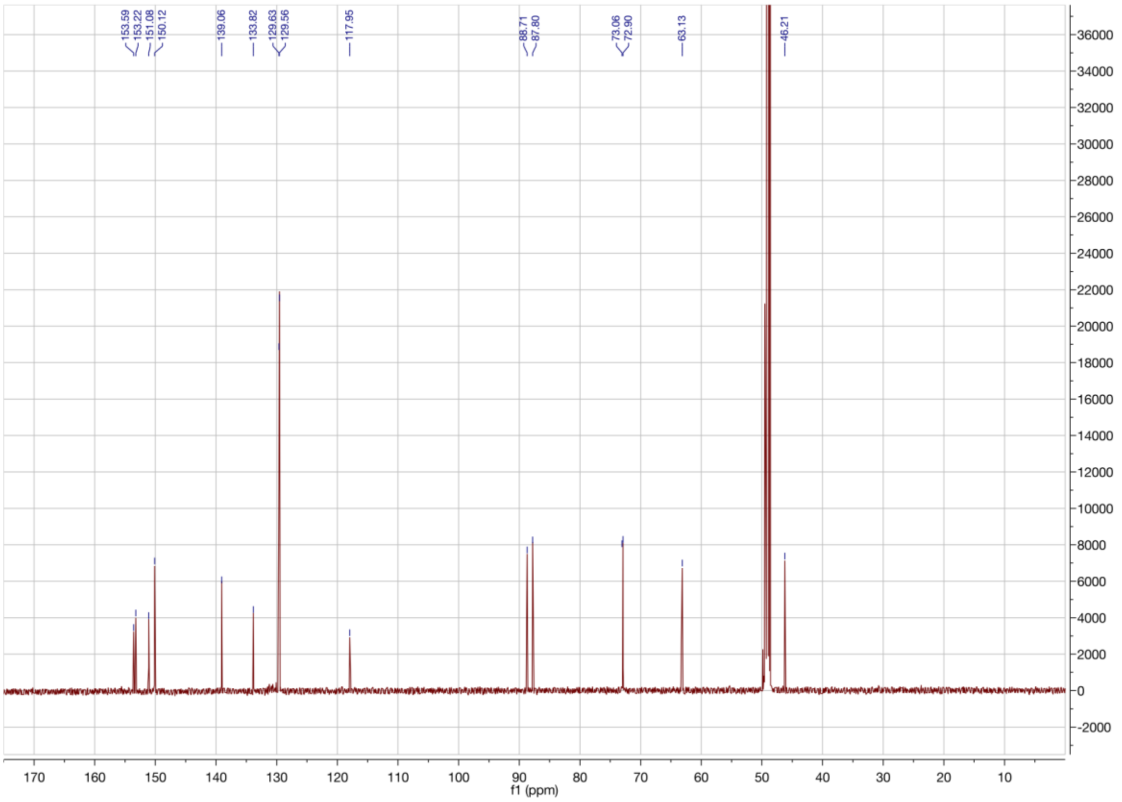

Ethyl(*E*)-3-((3a*R*,4*R*,6*R*,6a*R*)-6-(6-amino-9*H*-purin-9-yl)-2,2-dimethyltetrahydrofuro [3,4-*d*][1,3]dioxol-4-yl)acrylate (S2)

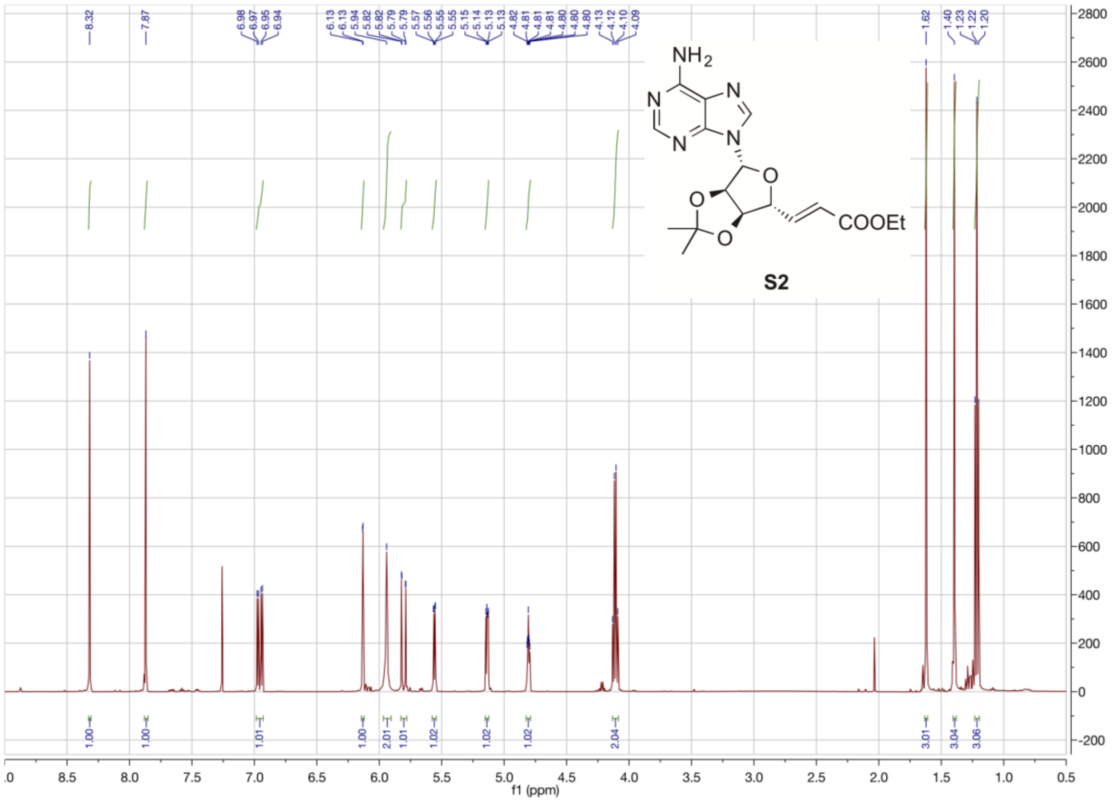

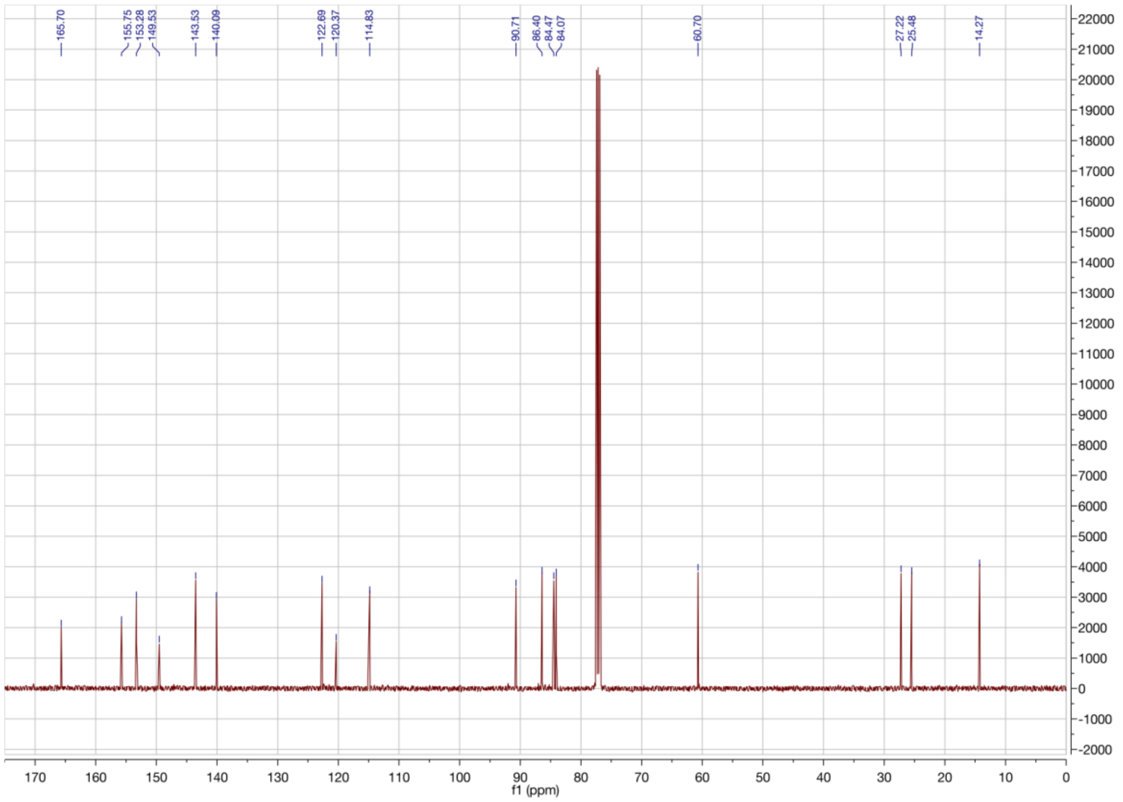

(*E*)-3-((3a*R*,4*R*,6*R*,6a*R*)-6-(6-Amino-9*H*-purin-9-yl)-2,2-dimethyltetrahydrofuro[3,4-*d*][1,3]dioxol-4-yl)prop-2-en-1-ol (S3)

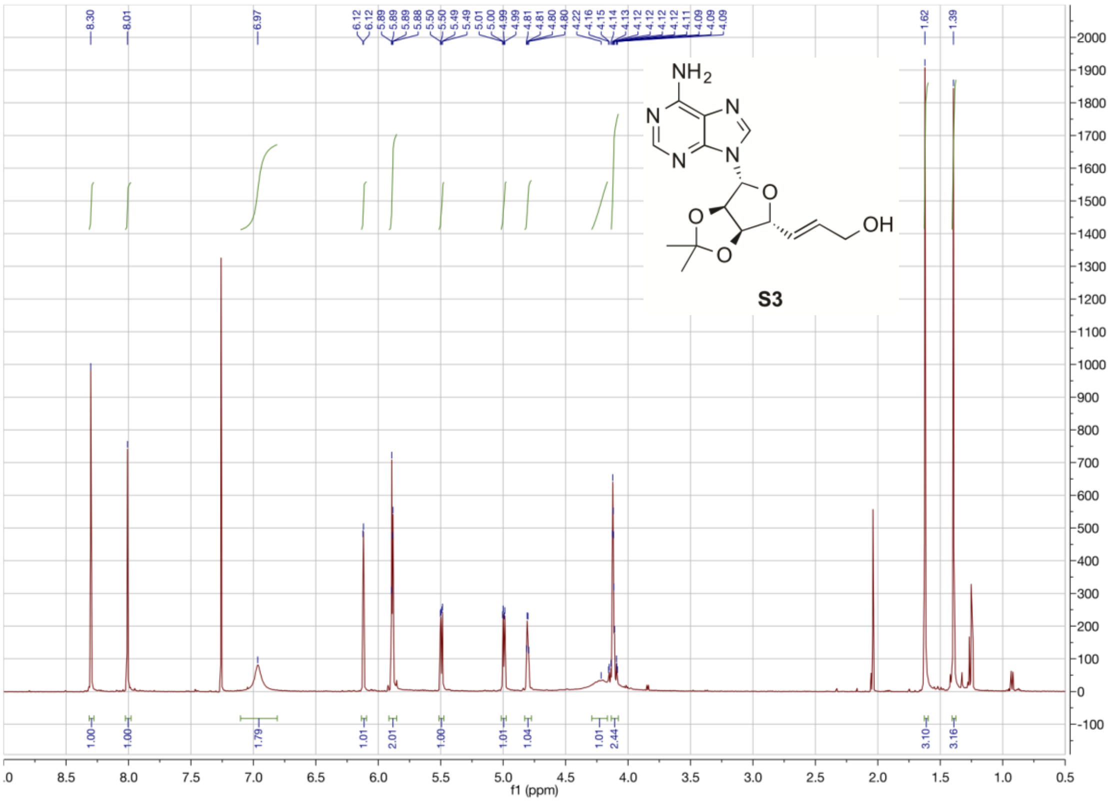

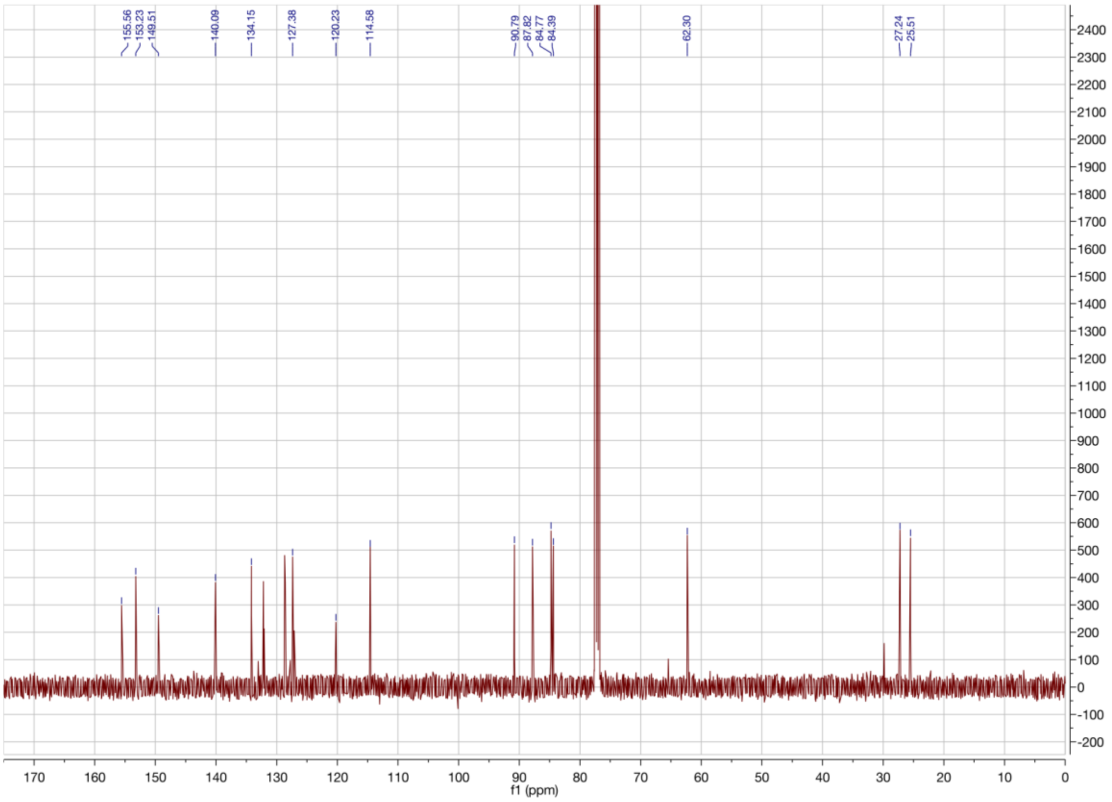

3-((3a*R*,4*R*,6*R*,6a*R*)-6-(6-Amino-9*H*-purin-9-yl)-2,2-dimethyltetrahydrofuro[3,4-*d*][1,3]dioxol-4-yl)propan-1-ol (5)

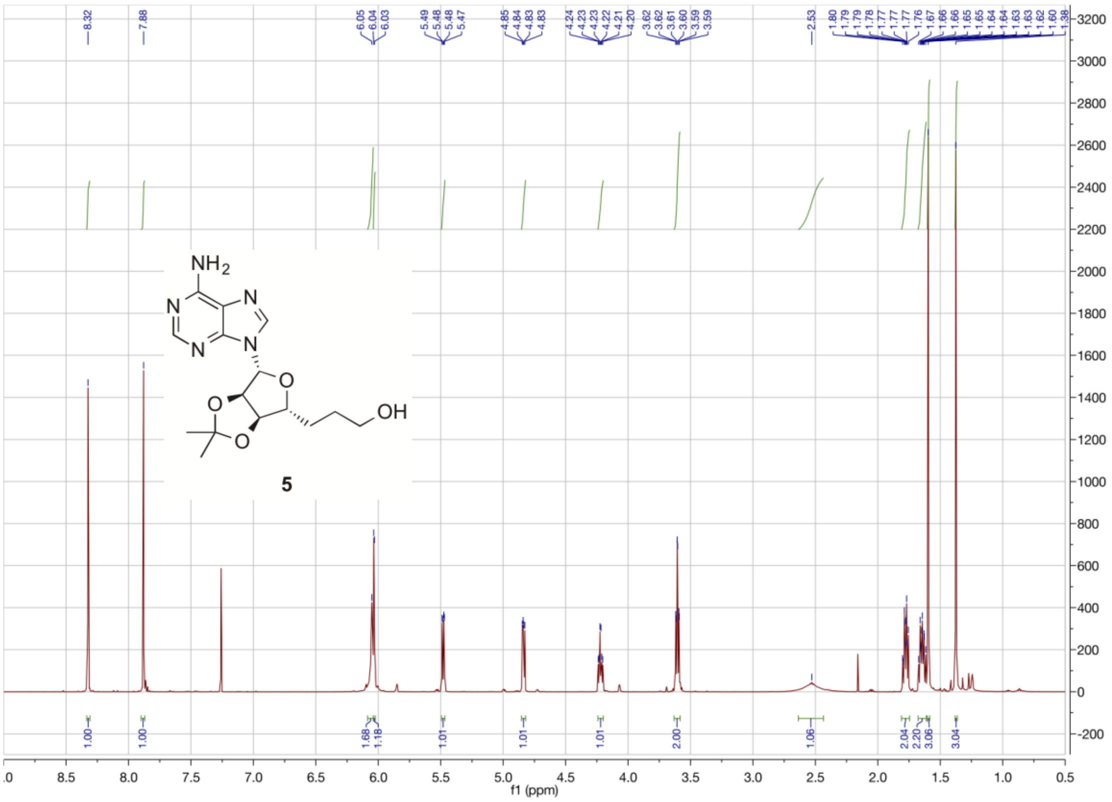

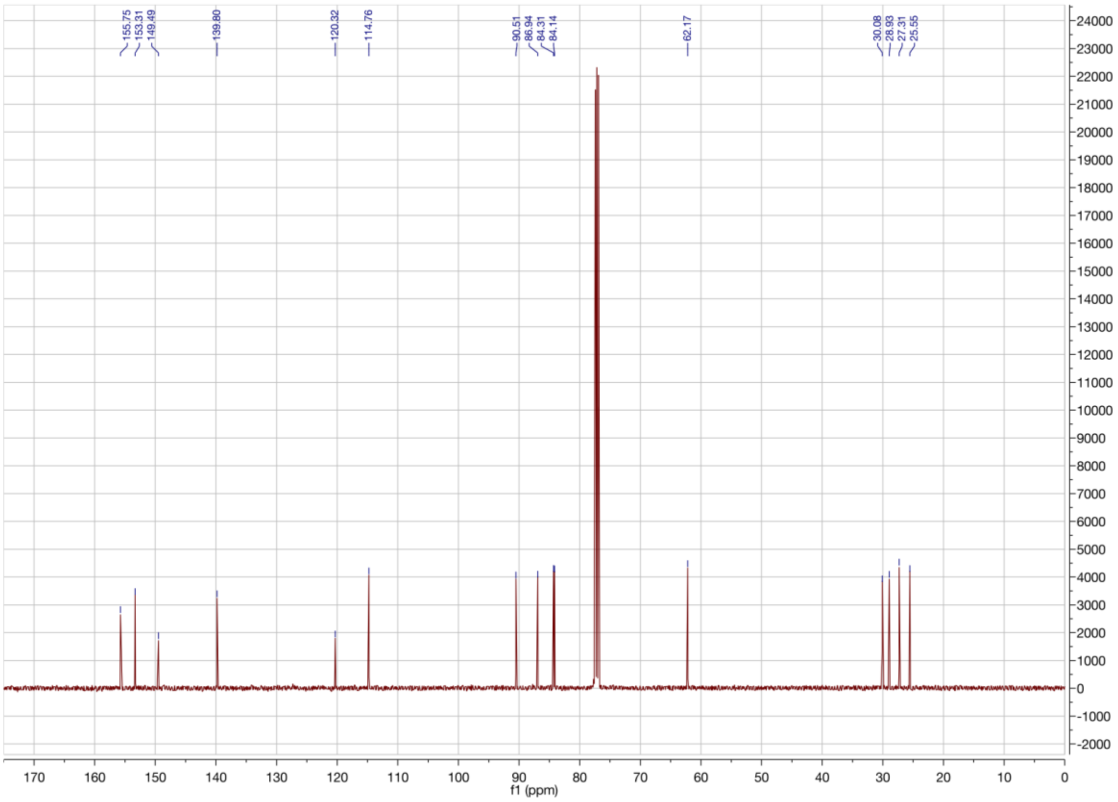

3-((3a*R*,4*R*,6*R*,6a*R*)-6-(6-Amino-8-bromo-9*H*-purin-9-yl)-2,2-dimethyltetrahydrofuro [3,4-*d*][1,3]dioxol-4-yl)propan-1-ol (6)

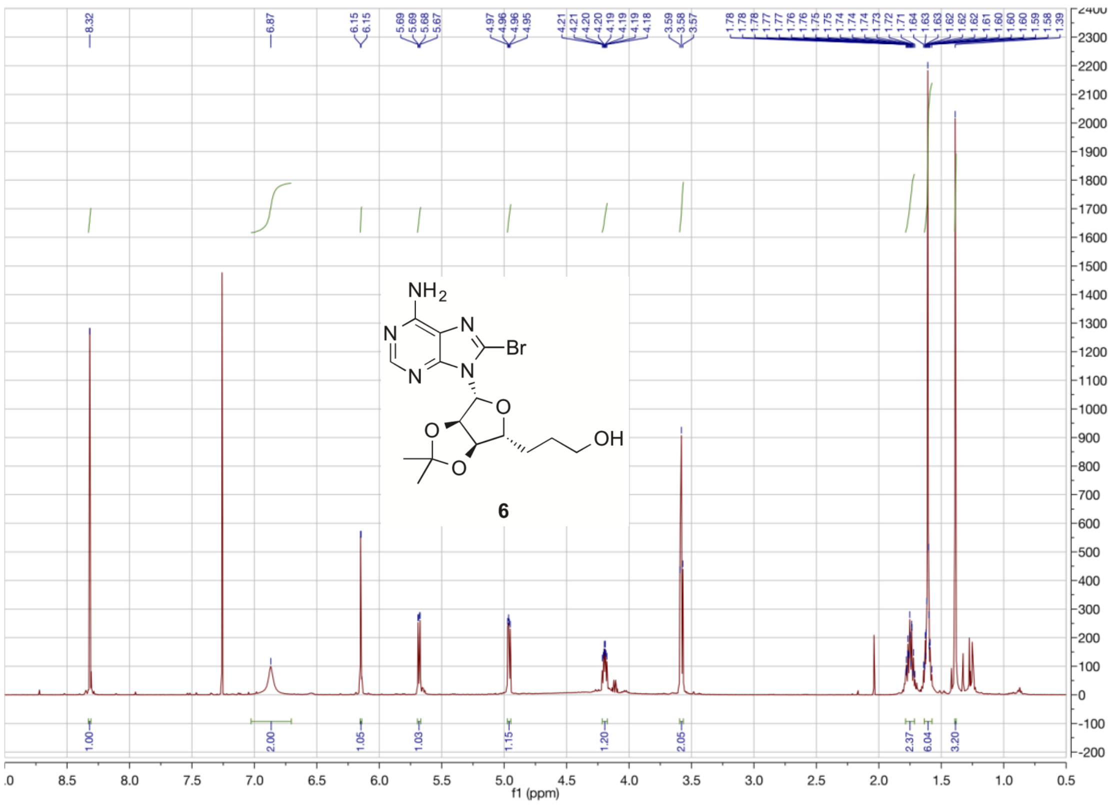

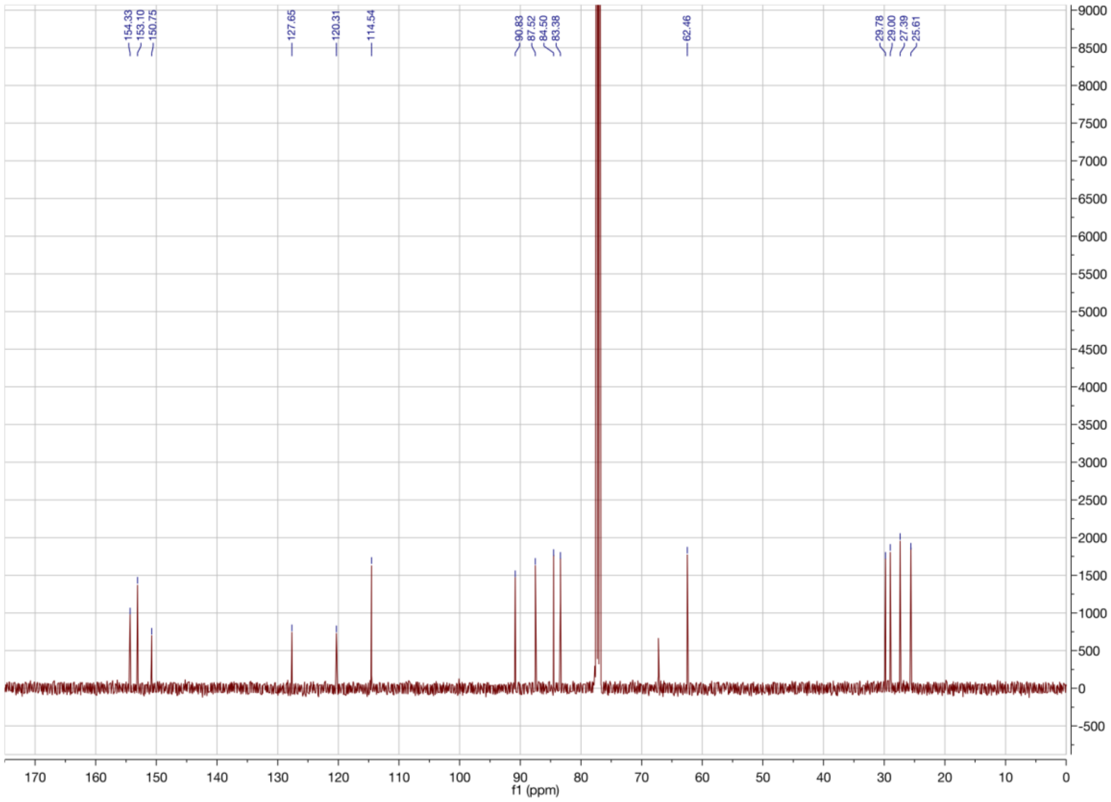

3-((3a*R*,4*R*,6*R*,6a*R*)-6-(6-Amino-8-((4-chlorobenzyl)amino)-9*H*-purin-9-yl)-2,2-dimethyltetrahydrofuro[3,4-*d*][1,3]dioxol-4-yl)propan-1-ol (7)

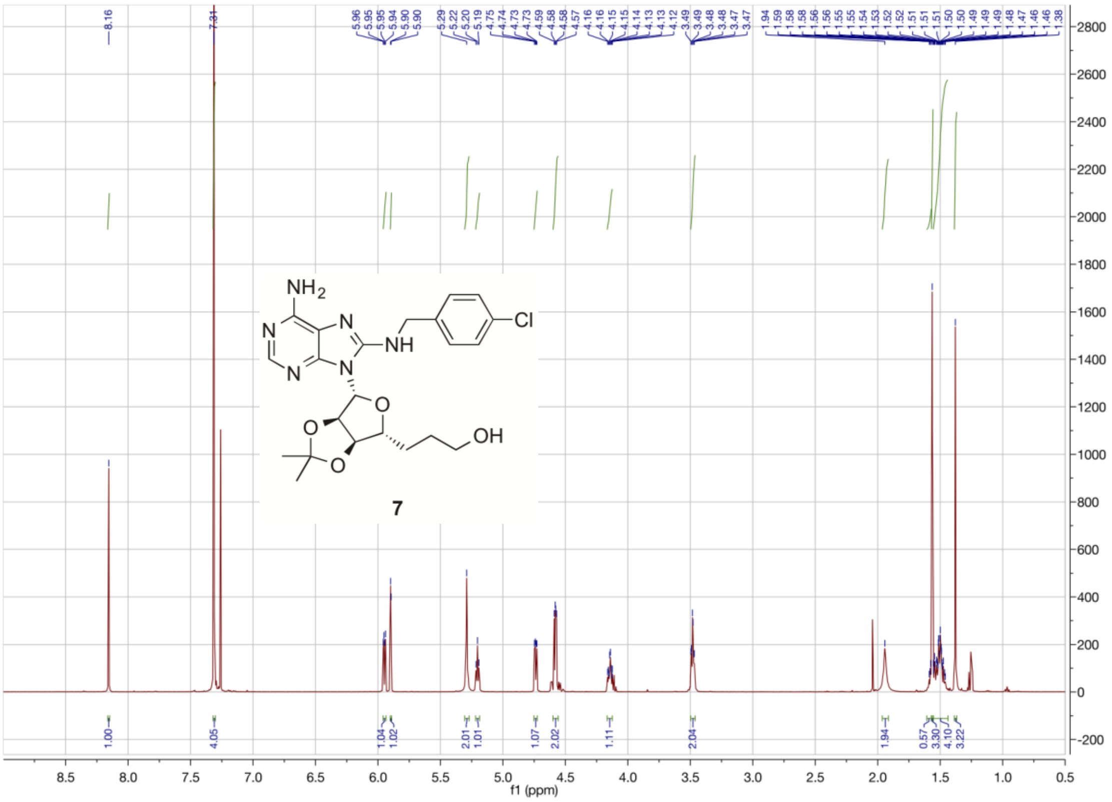

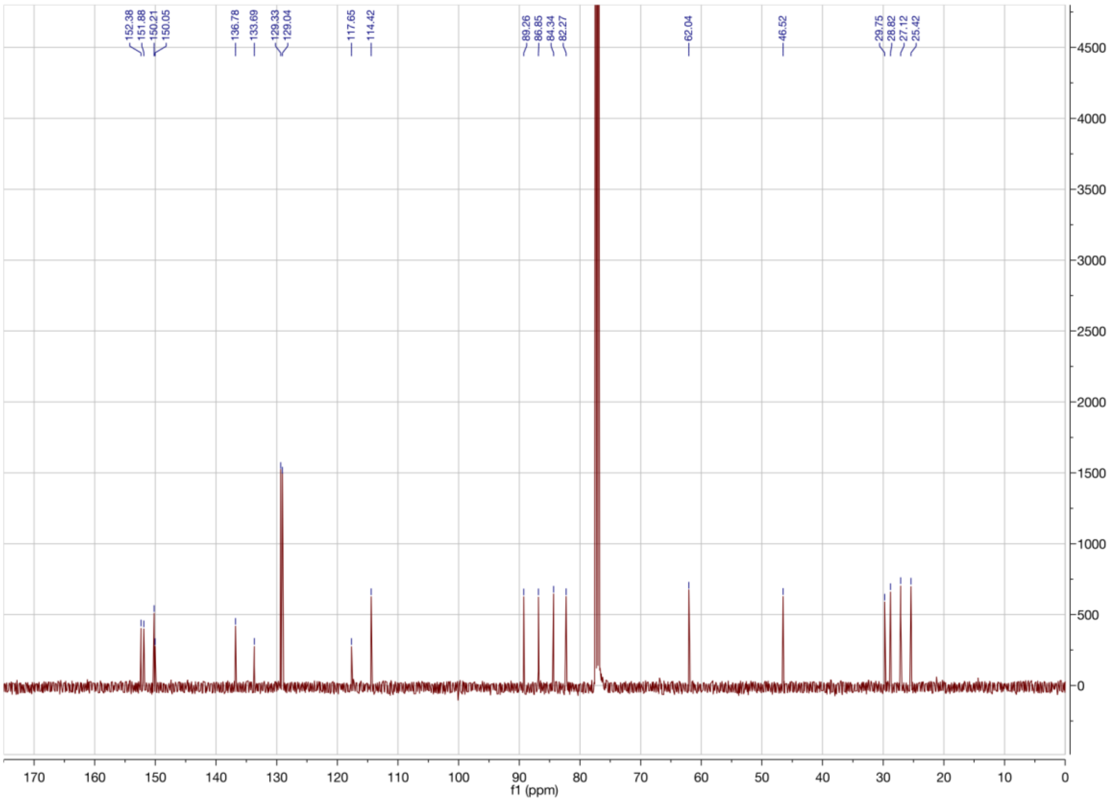

3-((3a*R*,4*R*,6*R*,6a*R*)-6-(6-Amino-8-((4-chlorobenzyl)amino)-9*H*-purin-9-yl)-2,2-dimethyltetrahydrofuro[3,4-*d*][1,3]dioxol-4-yl)propyl acrylate (10)

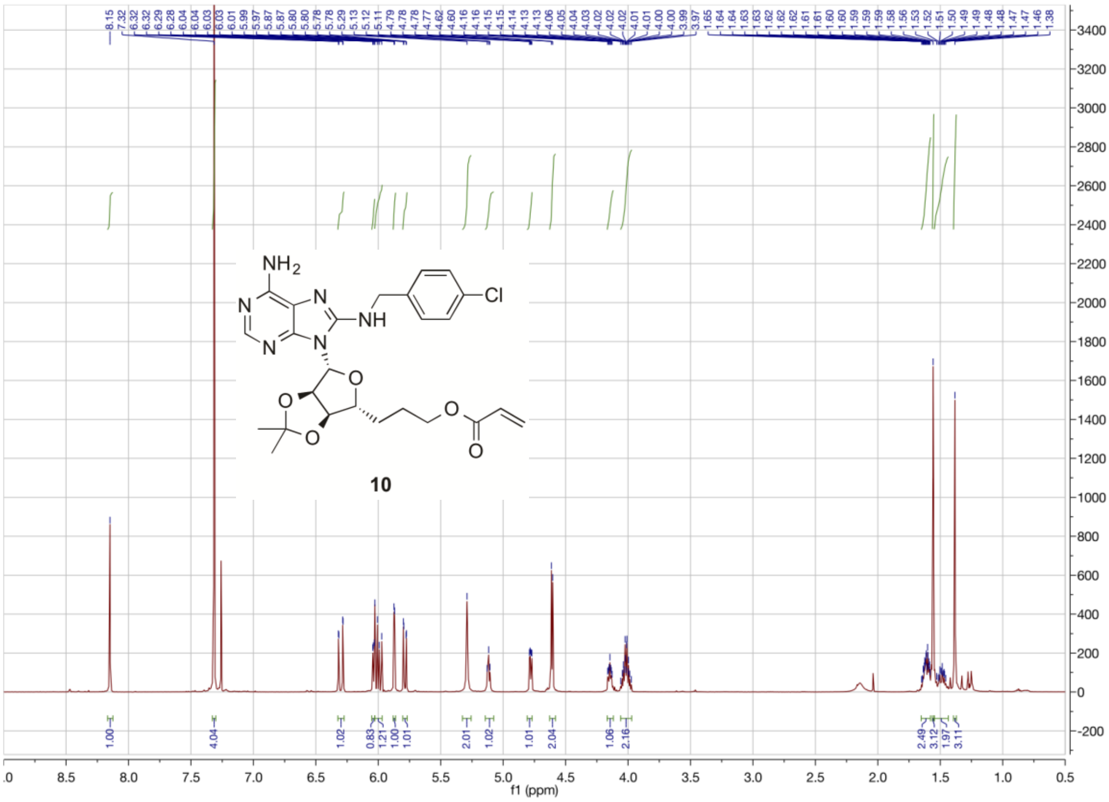

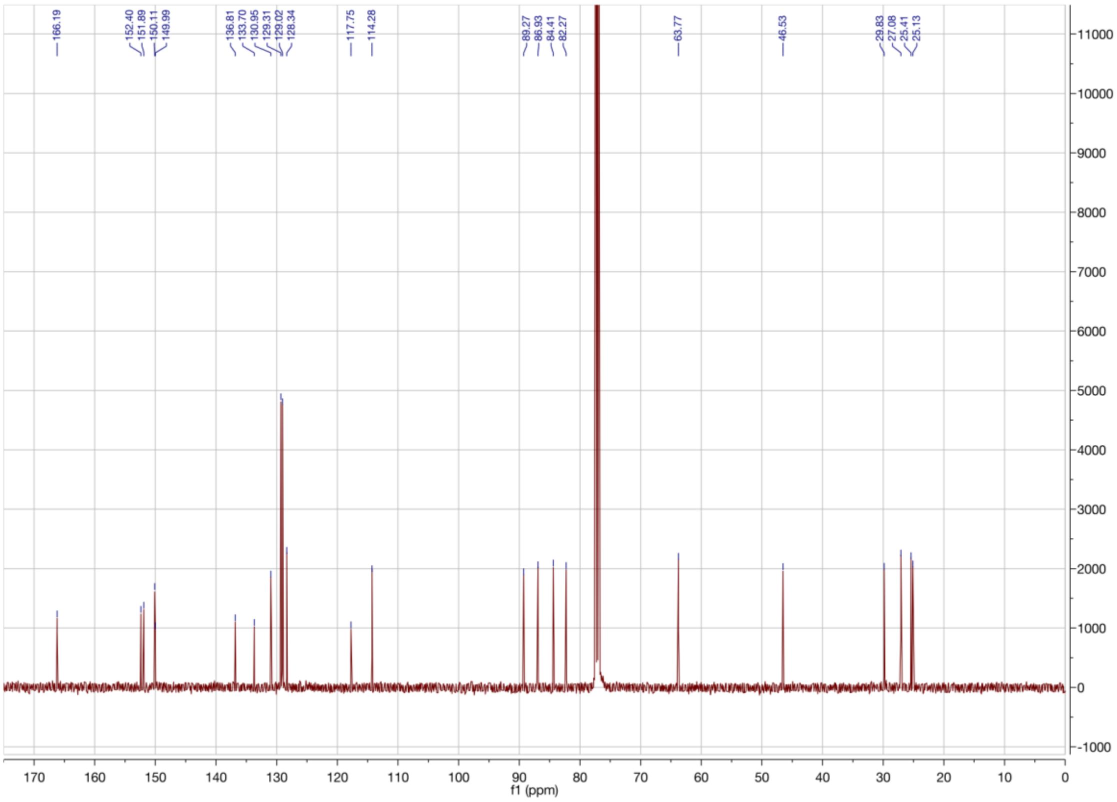

3-((2*R*,3*S*,4*R*,5*R*)-5-(6-Amino-8-((4-chlorobenzyl)amino)-9*H*-purin-9-yl)-3,4-dihydroxytetrahydrofuran-2-yl)propyl acrylate (8)

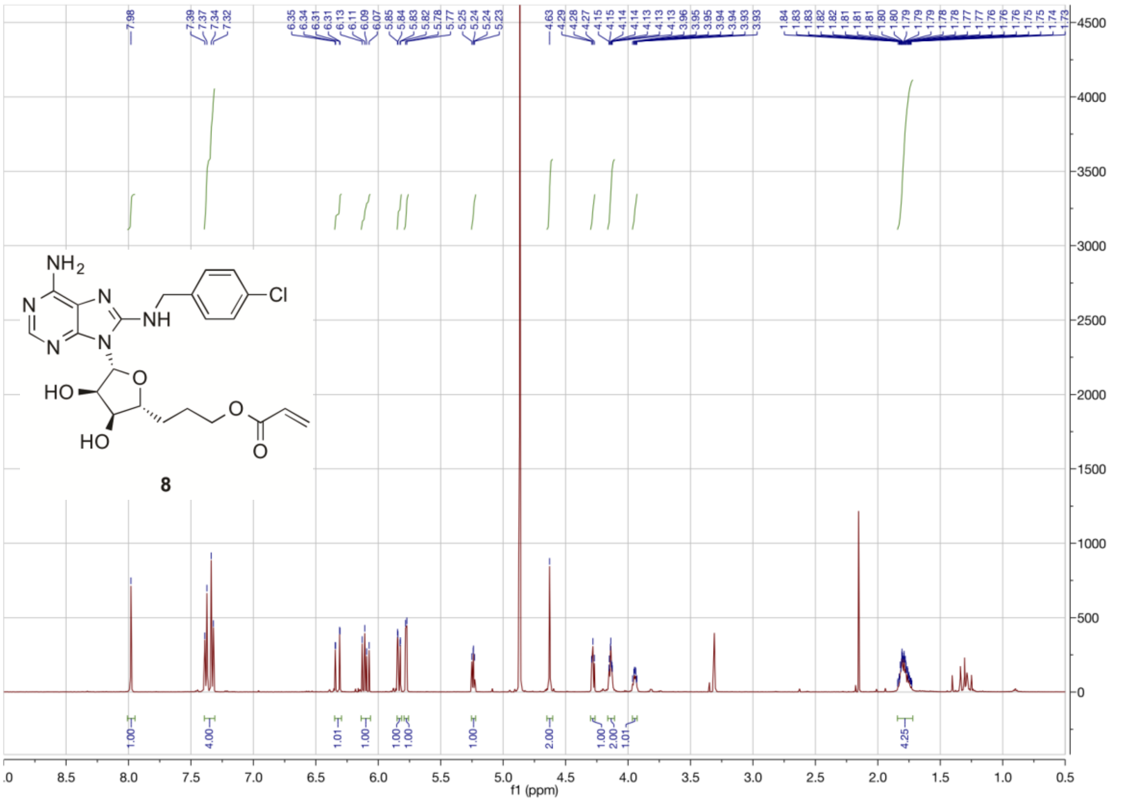

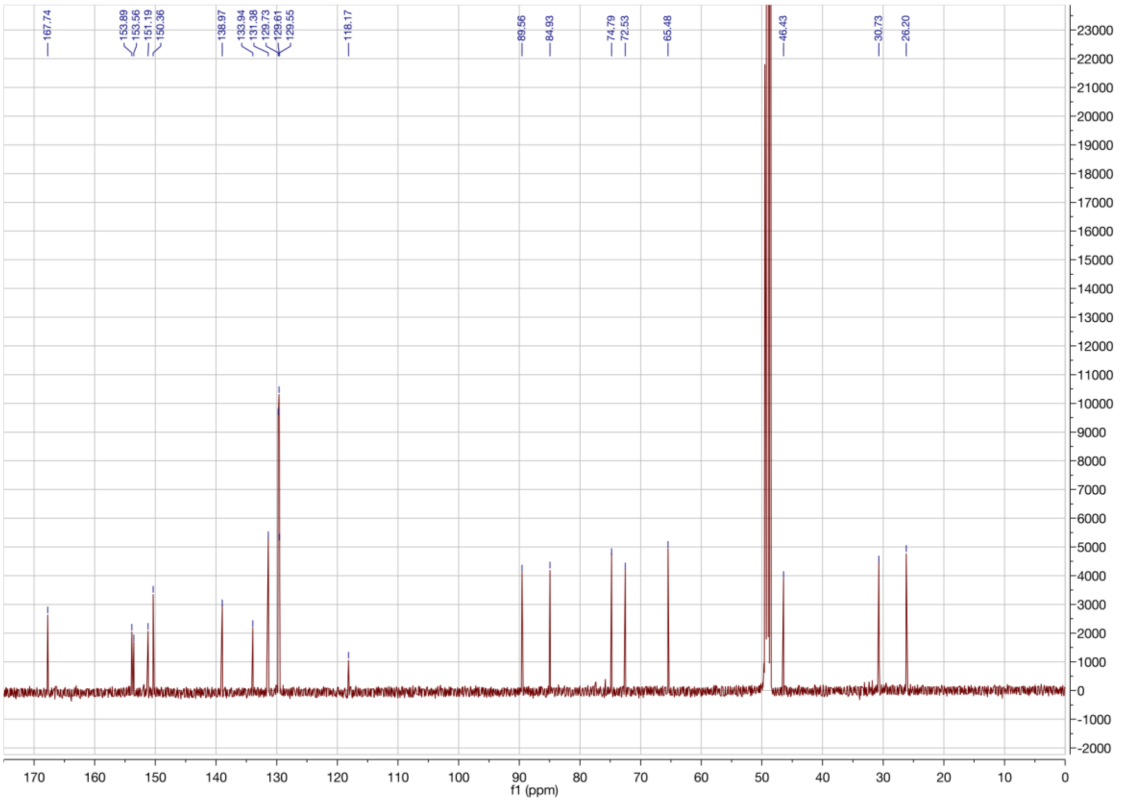

3-((3a*R*,4*R*,6*R*,6a*R*)-6-(6-Amino-8-((4-chlorobenzyl)amino)-9*H*-purin-9-yl)-2,2-dimethyltetrahydrofuro[3,4-*d*][1,3]dioxol-4-yl)propyl propionate (S4)

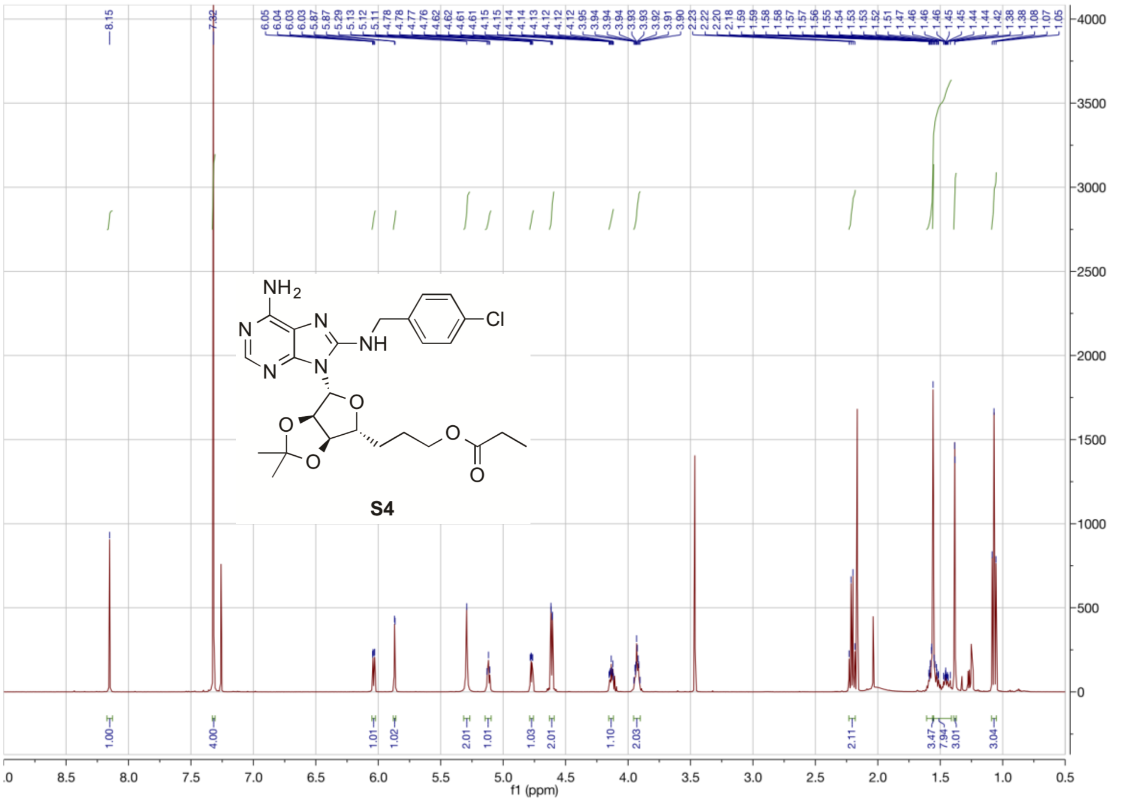

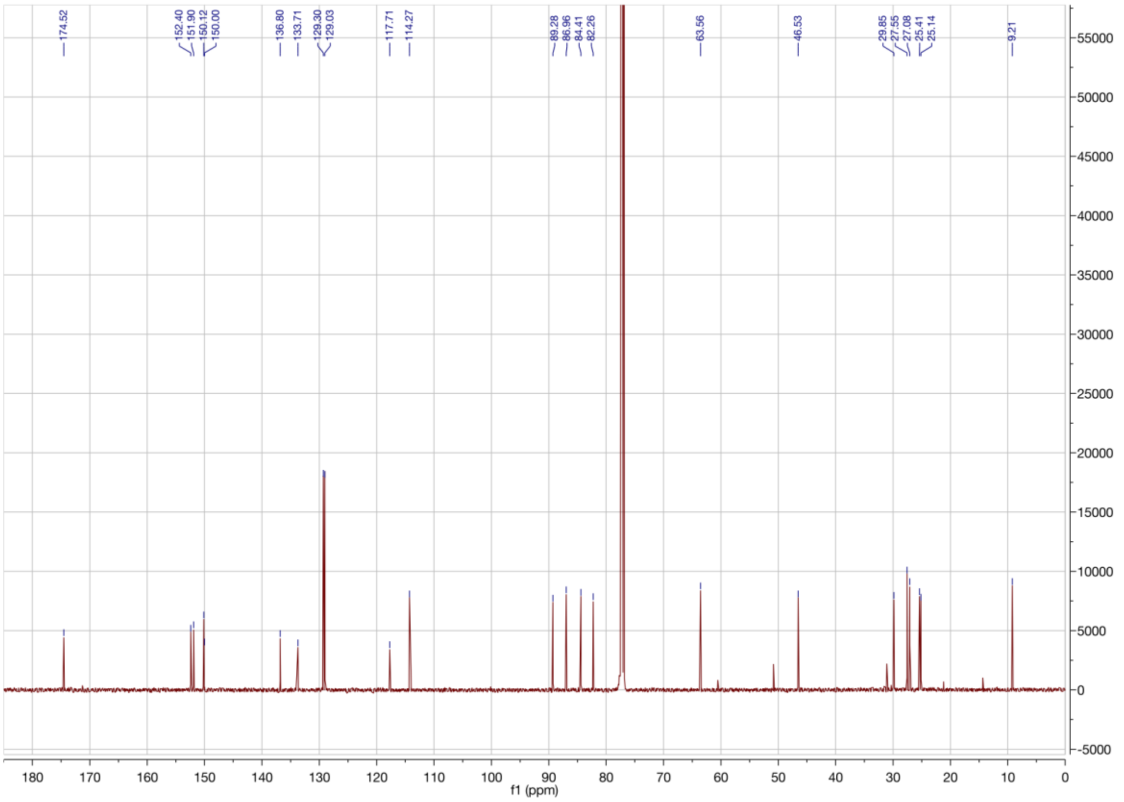

3-((2*R*,3*S*,4*R*,5*R*)-5-(6-Amino-8-((4-chlorobenzyl)amino)-9*H*-purin-9-yl)-3,4-dihydroxytetrahydrofuran-2-yl)propyl propionate (9)

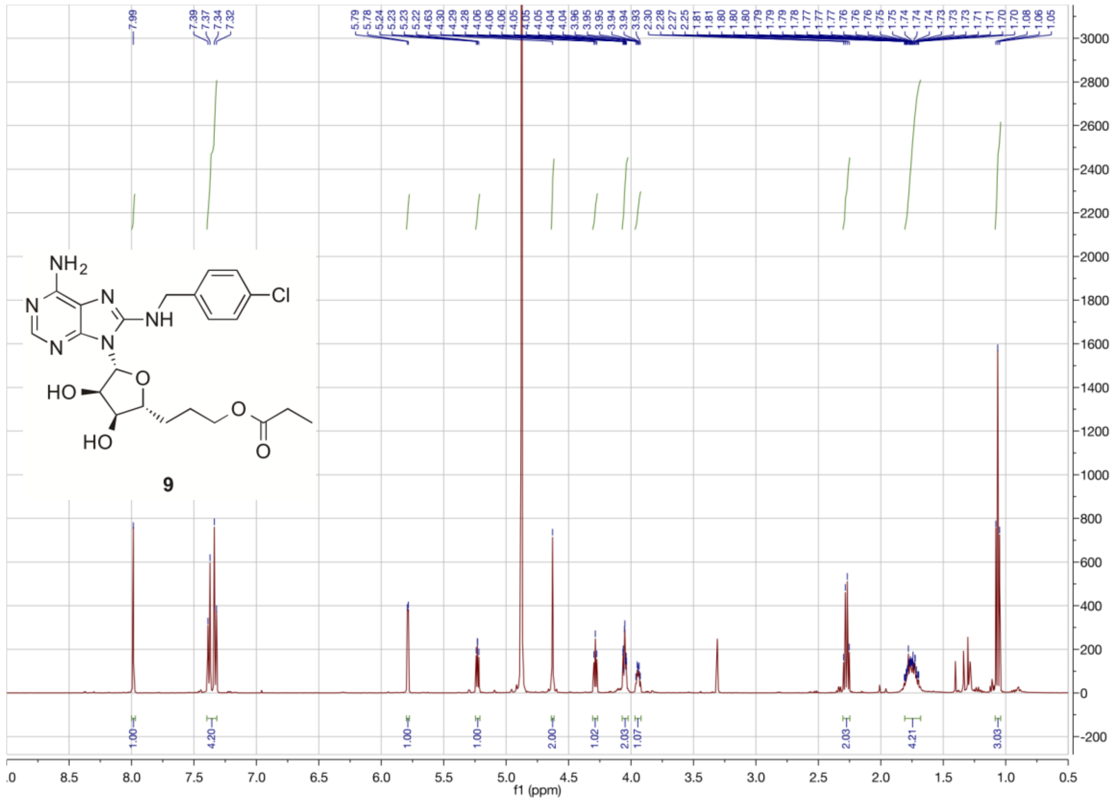

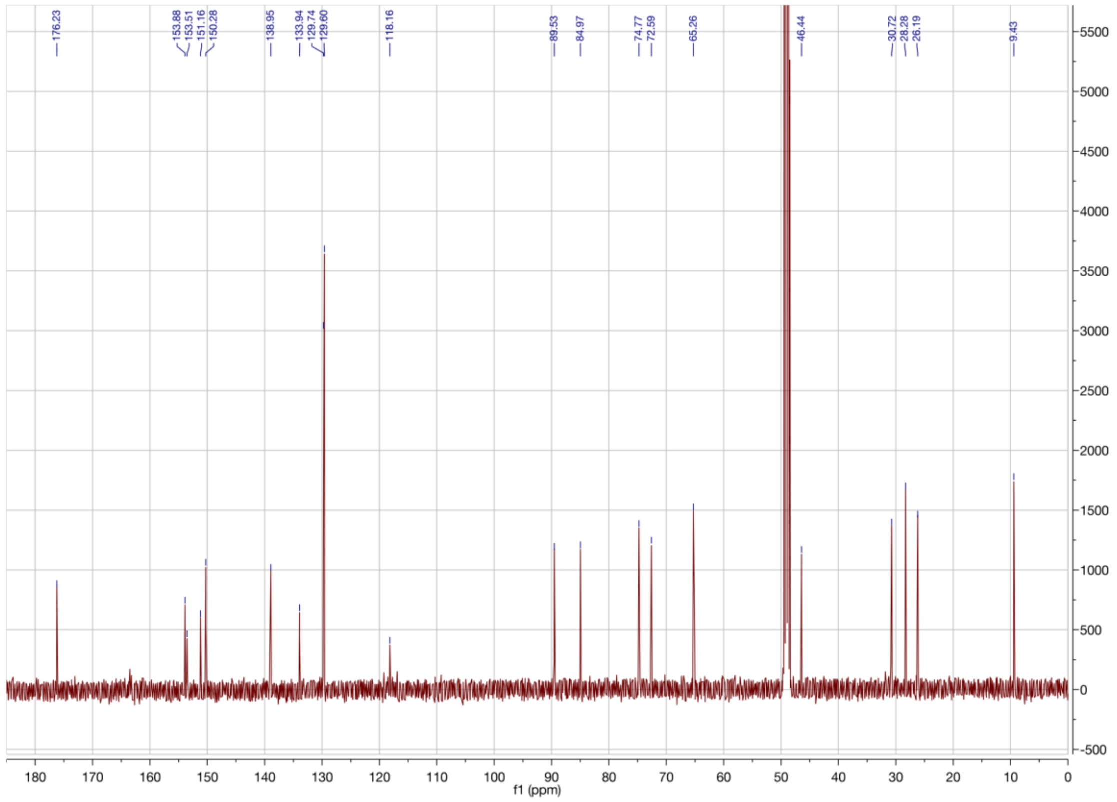

Protein Production and Purification Experimental

Purification of WT HSP72-NBD

The coding sequence for residues 1 to 380 HSPA1A (HSP72) was amplified by PCR from the IMAGE clone 3345864 using forward primer 5'-GATCGACATATGGCCAAAGCCGCGGCGATC-3' and its reverse complement. PCR products were inserted into the BamHI and SalI sites of pGEX-6P-1 vector (GE Healthcare), also encoding a glutathione-*S*-transferase (GST) tag cleavable with PreScission™ protease (GE Healthcare). BL21-AI™ cells (Invitrogen, Paisley, UK) transformed with the vector containing the HSPA1A-NBD gene were grown in Luria-Bertani (LB) medium to an optical density at 600 nm (OD_{600}) of 0.6 and induced with 0.5 mM isopropyl-β-D-1-thiogalactopyranoside (IPTG) and 0.2% (w/v) L-arabinose at 18 °C for 16 hours. Cells were harvested by centrifugation for 20 minutes at 5500 RPM and 4 °C using an Avanti centrifuge J-26XP (Beckman Coulter™, High Wycombe, UK) with a JLA 8.100 rotor. Cell pellets were resuspended in 3 volumes of lysis buffer consisting of 25 mM Tris, 50 mM NaCl, 2 mM EDTA, 5% (v/v) glycerol, 1 x cOmplete™ EDTA-free protease inhibitor (Roche, Basel, Switzerland), 12.5 U/mL Benzonase® nuclease (Merck Chemicals Ltd), 1 mg lysozyme, 1mM $MgCl_2$ at pH 7.5. Cells were lysed by sonication using a VibraCell™ VCX500 (Sonics & Materials Inc, Newtown, USA) with a 13 mm solid probe for 24 cycles of 5 second on, 55 second off, at 55% amplitude. Lysate was clarified by centrifugation for 40 minutes at 21,500 RPM at 4 °C using an Avanti centrifuge J-26XP (Beckman Coulter™) with a JA 25.50 rotor.

The supernatant was filtered using a 1.2 µm syringe filter (Sartorius Stedim, Germany), loaded onto a 5 mL GSTrap FF column (GE Healthcare, Chalfront St. Giles, UK) equilibrated in lysis buffer, washed with a wash buffer (25 mM Tris, 500 mM NaCl, 2 mM EDTA, 1 mM DTT, 5% (v/v) glycerol, pH 7.5), then eluted with an elution buffer (25 mM Tris, 50 mM NaCl, 2 mM EDTA, 1 mM DTT, 20 mM L-glutathione (reduced), pH 7.5). Fractions containing GST-tagged HSP72-NBD were pooled, followed by the addition of 5 U/mg PreScission™ protease (GE Healthcare) and incubation at 4 °C overnight. Subsequently, the sample was further purified using a Superdex 200 (26/60) size exclusion column (GE Healthcare) equilibrated in 25 mM Tris, 50 mM NaCl, 1 mM DTT, 5% (v/v) glycerol, pH 7.5, with the output connected to the GSTrap FF column. Fractions containing HSP72-NBD were pooled and further purified to remove contaminating nucleotides using a 6 mL Resource™ Q column (GE Healthcare) equilibrated in 25 mM Tris2 mM EDTA, 5% (v/v) glycerol, pH 7.5, then washed with the same buffer containing 500 mM NaCl. The pooled fractions were concentrated to 10 mL, then loaded onto a Superdex 200 (26/60) column (GE Healthcare) equilibrated in a buffer containing 25 mM Tris, 50 mM NaCl, 1 mM DTT, 5% (v/v) glycerol, pH 7.5. The removal of

contaminating nucleotides was monitored by measuring the ratio of absorbance at 260 nm and 280 nm (A_{260}/A_{280}) using a NanoDrop ND-1000 UV spectrophotometer (Thermofisher, Wilmington, USA). Samples with A_{260}/A_{280} below 0.6 were considered nucleotide free.

Purification of C17A, C267A, C306A and K56A HSP72-NBD mutants

The mutants were generated by site-directed mutagenesis using respective forward primers:
5'-GCACCACCTACTCCGCCGTGGGGGTGTTCC-3',
5'-GCTGCGCACCGCCGCCGAGAGGGCCAAG-3',
5'-GGTTCGAGGAGCTGGCCTCCGACCTGTTCC-3'
5'-CGGGGATGCGGCCGCGAACCAGGTGGCG-3' and their reverse complements. All HSP72-NBD mutants were expressed, lysed and purified as described for the WT HSP72-NBD. The successful introduction of the C17A, C267A, C306A and K56A mutations was confirmed at the protein level by mass spectrometry.

Fluorescence Polarization Assay Experimental

General experimental

The aqueous buffer contained 50 mM TRIS base pH 7.4, 150 mM KCl, 5 mM $CaCl_2$ and 0.1% (w/w) CHAPS. The assay was conducted using 384 Plus F ProxiPlates (PerkinElmer) with a final assay volume of 10 µL. Plates were centrifuged at 1000 rpm for 1 minute prior to incubation and read using a 2103 Envision Multilable Plate Reader. Excitation and emission wavelengths used were 620 nm and 535 nm, respectively. Fluorescence polarization was measured in units of millipolarization (mP). All experiments were performed in triplicate. Data were plotted and analysed using GraphPad Prism 6, graphical data represents the arithmetic mean ± curve fitting standard error of the mean for a single representative experiment.

K_D determination

To each well, 5 µL of probe molecule **S5** (20 nM in assay buffer) and increasing concentrations of HSP72-NBD protein (5 µL, two-fold dilution series) were added. Fluorescence polarization values for tracer control wells (10 nM probe **S5** in assay buffer only) were subtracted from each data point prior to data analysis. K_D determination was performed using non-linear regression analysis (GraphPad Prism 6, one site-specific binding model). pK_D values are quoted as geometric mean ± standard error of the mean from 3 independent experiments.

Competitive binding experiments

Compounds (0.2 µL at 50 x screening concentration in DMSO) were dispensed using an ECHO 550 Liquid Handler (Labcyte Inc.). To the corresponding wells was added, 5 µL of probe molecule **S5** (20 nM in assay buffer) and 5 µL of protein (two times their final concentration in assay buffer) to give a 50% bound fraction. Tracer controls (10 nM probe molecule **S5** only) and bound tracer controls (10 nM probe in presence of appropriate protein concentration) were included on each assay plate. IC_{50} determination was performed using non-linear least squares curve fitting (GraphPad Prism 6, log(inhibitor) vs. response – variable slop (four parameters)). K_i values were calculated using Huang's equation below. pK_i values are quoted as geometric mean ± standard error of the mean from 3 independent experiments.

Chemical structure of nucleotide-derived fluorescent probe, ATP-ATTO-488 (S5)

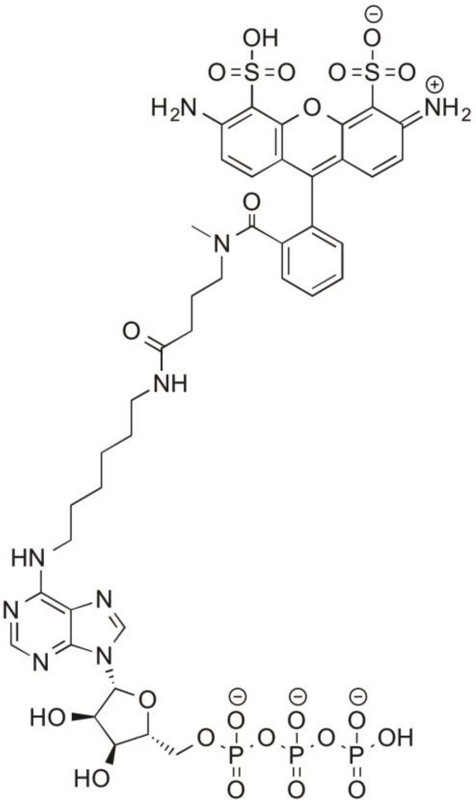

S5

N^6(6-Amino)hexyl-adenosine-5'-triphosphate, labelled with ATTO-488. Purchased from Jena Bioscience.

Huang's Equation describes the relationship between IC_{50} and K_i

Huang's equation was used to calculate K_i values from the measured IC_{50}s. See equation below:

$$IC_{50} = \left(\frac{f_0 K_d}{(1-f_0)(2-f_0)} + \frac{f_0 L_0}{2} \right) \left(\frac{K_i (2-f_0)}{K_d f_0} + 1 \right)$$

The equation states that the IC_{50} for a ligand that is competitive for binding with the assay probe is related to the binding affinity of the ligand (K_i), the bound fraction of the probe (f_0), the binding affinity of the probe (K_d) and the concentration of the probe (L_0). For competition experiments, it is recommended that a protein concentration giving a bound fraction between 0.5 and 0.8 be selected. A bound fraction below 0.5 will often result in an assay that is not statistically robust due to the decreased size of the binding window, however as the bound fraction approaches 1 the relationship between K_i and IC_{50} deviates from linear and the resolvable range of the assay decreases. For these reasons, a bound fraction of 0.5 was used for all assays.

Fluorescence Polarisation Assay Data

Representative Fluorescence Polarisation K_D determination curves with ATP-ATTO-488 (S5)

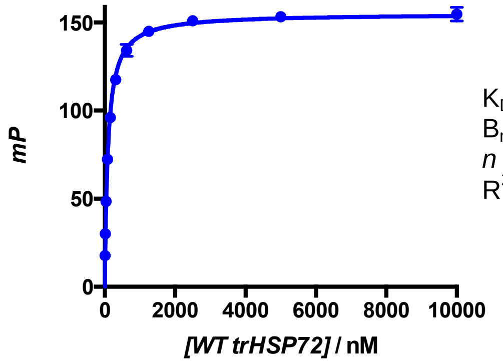

K_D = 90.1 ± 3.0 nM
B_{max} = 155.1 ± 1.1
n = 36
R^2 = 0.9959

WT HSP72-NBD
pK_D = 7.04 ± 0.03 (92 nM, *N* = 3)

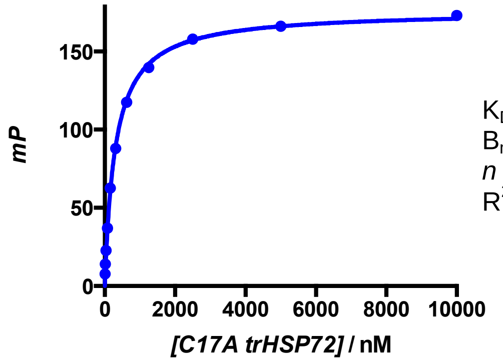

K_D = 298.8 ± 8.5 nM
B_{max} = 176.0 ± 1.2
n = 36
R^2 = 0.9978

C17A HSP72-NBD
pK_D = 6.58 ± 0.03 (260 nM, *N* = 3)

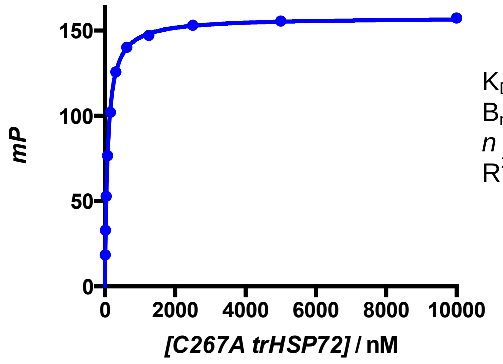

K_D = 79.9 ± 1.4 nM
B_{max} = 157.7 ± 0.6
n = 36
R^2 = 0.9988

C267A HSP72-NBD
pK_D = 7.09 ± 0.01 (81 nM, *N* = 3)

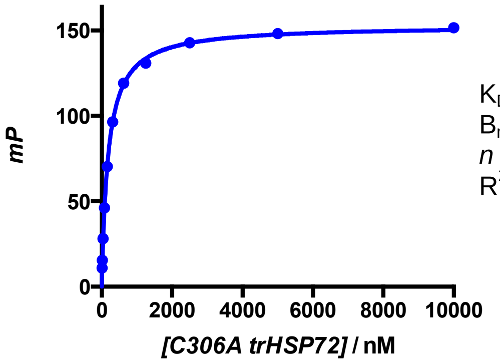

K_D = 181.4 ± 4.1 nM
B_{max} = 153.0 ± 0.8
n = 36
R^2 = 0.9984

C306A HSP72-NBD
pK_D = 6.74 ± 0.01 (180 nM, *N* = 3)

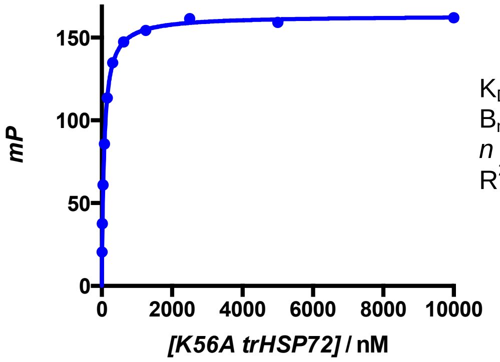

K_D = 67.4 ± 1.3 nM
B_{max} = 163.2 ± 0.6
n = 36
R^2 = 0.9985

K56A HSP72-NBD
pK_D = 7.180 ± 0.003 (66 nM, *N* = 3)

Representative Fluorescence Polarisation Assay IC_{50} curves

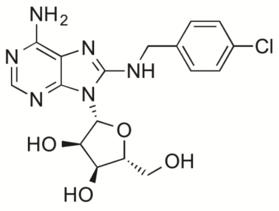

1

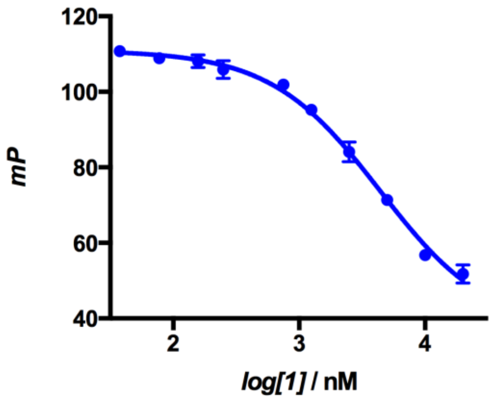

pIC_{50} = 5.36 ± 0.05 (4.4 μM)
Top = 111.0 ± 0.9
Bottom = 36.7 ± 2.8
n = 30
R^2 = 0.9850

pK_i = 5.72 ± 0.03 (1.9 μM, *N* = 3)

Fluorescence Polarisation Assay Time-Dependence Curves

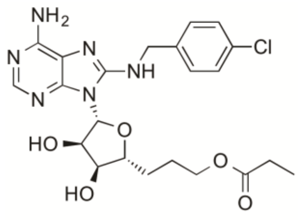

9

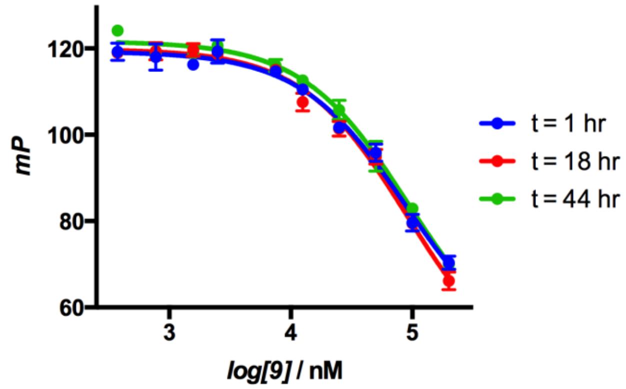

t =1 hr
pIC_{50} = 4.06 ± 0.08 (87.8 μM)
pK_i = 4.38 (41.8 μM)
Top = 119.3 ± 0.9
Bottom = 48.1 ± 5.8
n = 30
R^2 = 0.9689

t = 18 hr
pIC_{50} = 4.04 ± 0.07 (90.9 μM)
pK_i = 4.36 (43.3 μM)
Top = 119.9 ± 0.8
Bottom = 42.4 ± 5.4
n = 30
R^2 = 0.9782

t = 44 hr
pIC_{50} = 4.03 ± 0.07 (92.3 μM)
pK_i = 4.36 (43.9 μM)
Top = 121.7 ± 0.8
Bottom = 46.4 ± 5.4
n = 30
R^2 = 0.9769

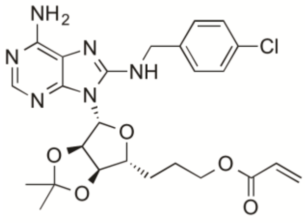

10

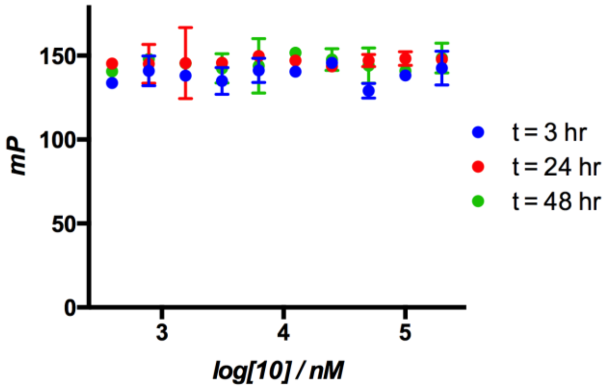

10 *shows no displacement of the ATP-probe and no time-dependent decrease in apparent* IC_{50}.

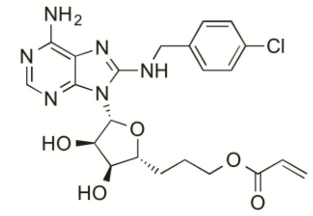

8

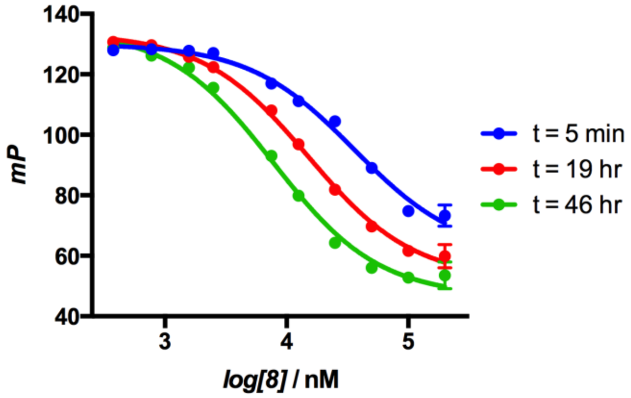

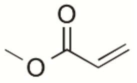

S6

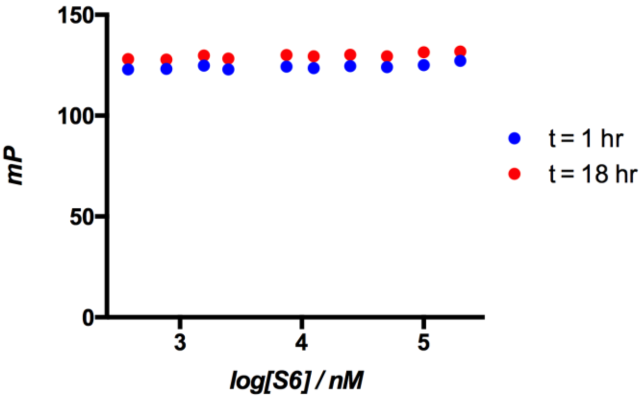

*Methyl acrylate **S6** shows no displacement of the ATP-probe and no time-dependent decrease in apparent IC_{50}.*

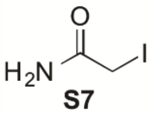

S7

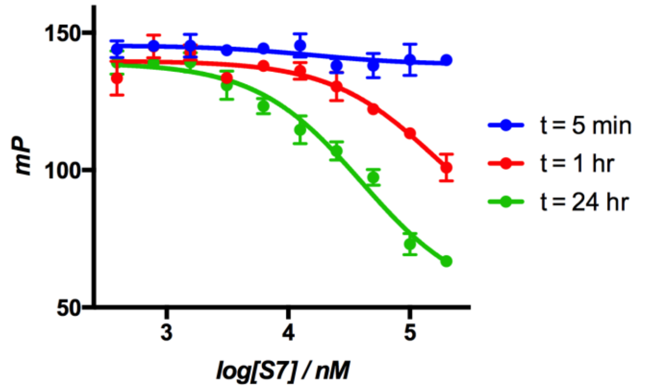

<u>t = 24 hr</u>
Apparent pIC_{50} = 4.40 ± 0.10 (40.1 μM)
Top = 139.1 ± 2.1
Bottom = 52.2 ± 6.2
n = 30
R^2 = 0.9447

*Iodoacetamide **S7** shows no initial displacement of the ATP-probe, however does show a time-dependent decrease in apparent IC_{50}.*

HSP72-NBD Mutant Fluorescence Polarisation time dependence

C17A HSP72-NBD + TCI (8)

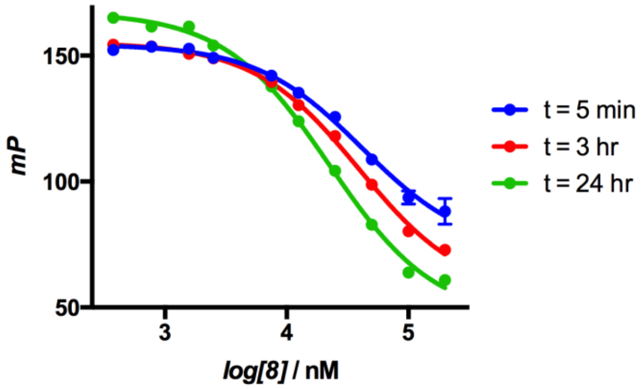

<u>t = 5 min</u>
pIC_{50} = 4.38 ± 0.05 (41.6 μM)
pK_i = 4.69 (20.4 μM)
Top = 154.5 ± 1.1
Bottom = 72.2 ± 3.3
n = 30
R^2 = 0.9825

<u>t = 3 hr</u>
pIC_{50} = 4.40 ± 0.02 (39.4 μM)
pK_i = 4.71 (19.3 μM)
Top = 155.2 ± 0.6
Bottom = 54.6 ± 1.8
n = 30
R^2 = 0.9964

<u>t = 24 hr</u>
pIC_{50} = 4.65 ± 0.02 (22.6 μM)
pK_i = 4.96 (11.1 μM)
Top = 167.2 ± 1.0
Bottom = 45.4 ± 1.8
n = 30
R^2 = 0.9958

C267A HSP72-NBD + TCI (8)

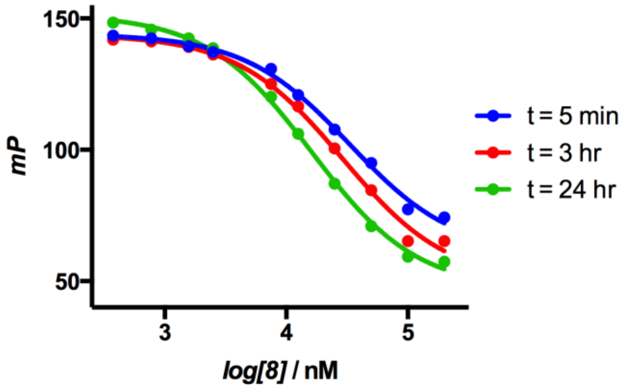

t = 5 min
pIC_{50} = 4.48 ± 0.03 (33.2 μM)
pK_i = 4.80 (15.8 μM)
Top = 144.1 ± 0.8
Bottom = 60.1 ± 2.0
n = 30
R^2 = 0.9922

t = 3 hr
pIC_{50} = 4.54 ± 0.03 (28.9 μM)
pK_i = 4.86 (13.8 μM)
Top = 143.9 ± 0.9
Bottom = 49.6 ± 2.0
n = 30
R^2 = 0.9930

t = 24 hr
pIC_{50} = 4.79 ± 0.02 (16.2 μM)
pK_i = 5.11 (7.7 μM)
Top = 151.5 ± 0.8
Bottom = 46.8 ± 1.2
n = 30
R^2 = 0.9970

C306A HSP72-NBD + TCI (8)

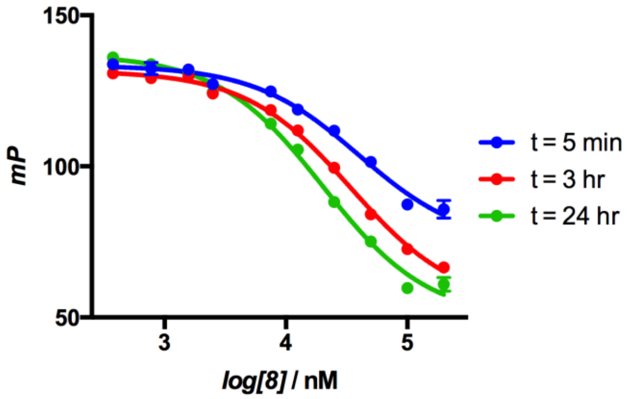

t = 5 min
pIC_{50} = 4.40 ± 0.06 (39.9 μM)
pK_i = 4.71 (19.5 μM)
Top = 133.4 ± 0.9
Bottom = 74.0 ± 2.7
n = 30
R^2 = 0.9762

t = 3 hr
pIC_{50} = 4.46 ± 0.03 (34.4 μM)
pK_i = 4.78 (16.8 μM)
Top = 131.7 ± 0.7
Bottom = 53.9 ± 1.7
n = 30
R^2 = 0.9936

t = 24 hr
pIC_{50} = 4.69 ± 0.04 (20.3 μM)
pK_i = 5.01 (9.9 μM)
Top = 137.0 ± 1.0
Bottom = 49.5 ± 1.8
n = 30
R^2 = 0.9915

K56A HSP72-NBD + TCI (8)

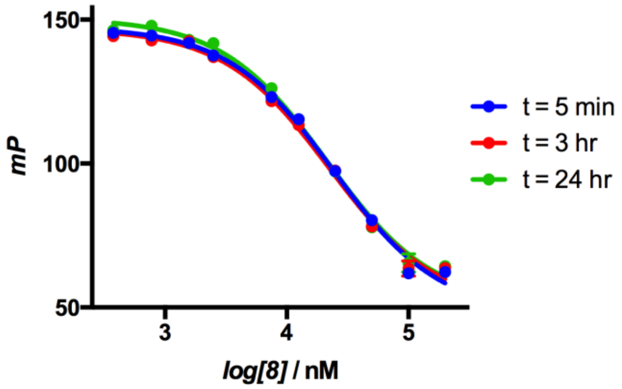

t = 5 min
pIC_{50} = 4.62 ± 0.04 (26.9 μM)
pK_i = 4.94 (11.4 μM)
Top = 148.8 ± 1.0
Bottom = 55.2 ± 2.1
n = 30
R^2 = 0.9916

t = 3 hr
pIC_{50} = 4.65 ± 0.04 (22.2 μM)
pK_i = 4.98 (10.5 μM)
Top = 146.9 ± 1.2
Bottom = 50.2 ± 2.2
n = 30
R^2 = 0.9903

t = 24 hr
pIC_{50} = 4.67 ± 0.04 (21.6 μM)
pK_i = 4.99 (10.2 μM)
Top = 150.5 ± 1.1
Bottom = 50.8 ± 2.1
n = 30
R^2 = 0.9916

All 3 Cys-Ala mutants still show time-dependent decrease in apparent IC_{50}s. The K56A mutant no longer shows a time-dependent drop in apparent IC_{50}.

Tool compound Fluorescence Polarisation curves

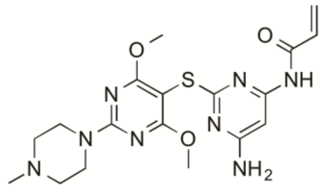

2

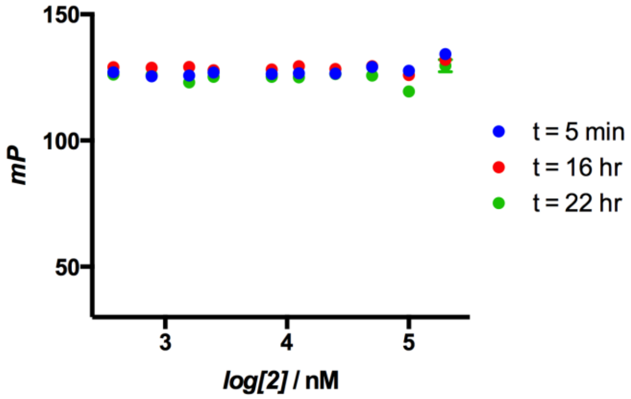

*YK5 **2** shows no displacement of the ATP-probe and no time-dependent decrease in apparent IC_{50}.*

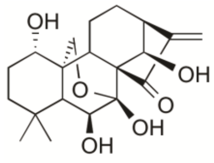

3

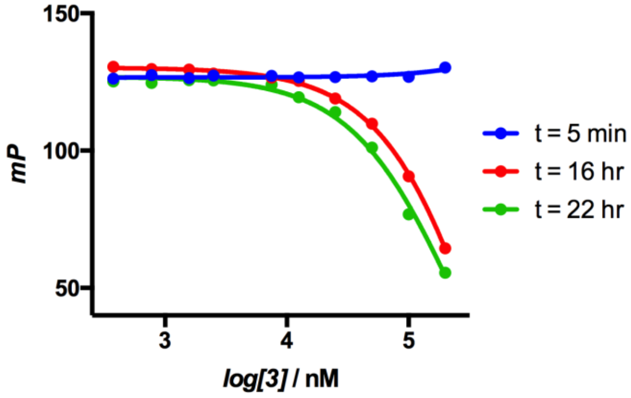

*Oridonin **3** shows no initial displacement of ATP-probe, however does show a time-dependent decrease in apparent IC_{50} presumably due to non-specific reactivity.*

HSP72 Protein Mass Spectrometry Experimental

LC-MS for intact proteins

HSP72-NBD (2.3 µM) in buffer (50 mM TRIS base pH 7.4, 150 mM KCl, 5 mM $CaCl_2$) was mixed with compound (200 µM from 10 mM DMSO stock) and incubated at 21 °C prior to analysis. The total reaction volume was 100 µL. Reaction was stopped by taking sample using auto-sampler. 1 µL injections of the sample were made onto a Security Guard C8 column cartridge (4 x 3 mm, AJO-4290, Phenomenex). Samples were incubated at 21 °C in a G1367B auto-sampler (Agilent) with G1330B thermostat module prior to injection. Chromatographic separation at 21 °C was carried out using an Agilent 1200 Series HPLC over a 1 minute gradient elution of 95:5 to 5:95 water and acetonitrile (both modified with 0.1% formic acid) at a flow rate of 0.5 mL/min. The post column eluent flow was infused into an Agilent 6520 Series qToF mass spectrometer fitted with a dual ESI ionization source. An initial divert to waste was used to aid desalting. LC eluent and nebulizing gas was introduced into the grounded nebulizer with spray direction orthogonal to the capillary axis. The aerosol was dried by heated gas (10 L/min of nitrogen at 350 °C, 50 psi), producing ions by ESI. Ions entered the transfer capillary along which a potential difference of 4 kV was applied. The fragmentor voltage was set at 190 V and skimmer at 65 V. Signal was optimized by AutoTune.m. Profile mass spectrometry data were acquired in positive ionization mode over a scan range of m/z 650-2000 (scan rate 1.0) with reference mass correction at m/z 922.009798 hexakis(1H,1H,3H-perfluoropropoxy)phosphazene. Raw data were processed using Agilent MassHunter Qualitative Analysis B.06.00 and graphs made using GraphPad Prism 6.

LC-MS/MS for Trypsin-digested proteins

Solvents were purchased from Rathburn (Walkerburn, UK). All other reagents were purchased from Sigma (Poole, UK) unless otherwise stated. HSP72-NBD (2.3 µM) in buffer (50 mM TRIS base pH 7.4, 150 mM KCl, 5 mM $CaCl_2$) was mixed with TCI **8** (200 µM from 10 mM DMSO stock) and incubated at 21 °C for 72 hours prior to analysis. A sample minus TCI **8** was also incubated at 21 °C for 72 hours. An aliquot corresponding to 10 pmol of protein of each sample were diluted 2-fold with 50 mM triethylammonium bicarbonate/5% acetonitrile. These were then reduced with 5 mM TCEP, alkylated with 10 mM chloroacetamide, and the proteins digested by addition of 20 ng trypsin and incubation for 1 h at 37 °C, followed by a second addition of 20 ng trypsin and incubation for a further 3 h at 37 °C. The digestion solution was purified in its entirety by C18 ZipTip cleanup and dried *in*

vacuo. The dried eluate was reconstituted in 10 uL of 1% acetonitrile/0.1% formic acid for LC-MS/MS.

Reversed phase chromatography was performed using an HP1200 platform (Agilent, Wokingham, UK). Approximately 50% of the digested sample was analysed as a 6 µL injection. Peptides were resolved on a 75 µm I.D. 15 cm C18 packed emitter column (3 µm particle size; NIKKYO TECHNOS CO. LTD, Tokyo, Japan) over 30 min using a linear gradient of 96:4 to 50:50 buffer A:B (buffer A: 1% acetonitrile/3% dimethyl sulfoxide/0.1% formic acid; buffer B: 80% acetonitrile/3% dimethyl sulfoxide/0.1% formic acid) at 250 nL/min. DMSO was used based on the methods of Hahne et al.[1] Peptides were ionised by electrospray ionisation using 1.8 kV applied immediately pre-column via a microtee built into the nanospray source. Sample was infused into an LTQ Velos Orbitrap mass spectrometer (Thermo Fisher Scientific, Hemel Hempstead, UK) directly from the end of the tapered tip silica column (6-8 µm exit bore). The ion transfer tube was heated to 275 °C and the S-lens set to 60%. MS/MS were acquired using data dependent acquisition based on a full 30,000 resolution FT-MS scan with preview mode disabled and internal lock mass calibration against ion 401.922718. The top 10 most intense ions were fragmented by collision-induced dissociation and analysed using enhanced ion trap scans. Precursor ions with unknown or single charge states were excluded from selection. Automatic gain control was set to 1,000,000 for FT-MS and 30,000 for IT-MS/MS, full FT-MS maximum inject time was 500 ms and normalized collision energy was set to 35% with an activation time of 10 ms. Wideband activation was used to co-fragment precursor ions undergoing neutral loss of up to -20 *m/z* from the parent ion, including loss of water/ammonia. MS/MS was acquired for selected precursor ions with a single repeat count acquired after 5 s delay followed by dynamic exclusion with a 10 ppm mass window for 10 s based on a maximal exclusion list of 500 entries.

Raw MS/MS data was compiled and interrogated against the swissprot 2011_01 human database, customised to include the HSP72-NBD construct sequence, using Proteome Discoverer v1.4 and Mascot v2.3. The Mascot search parameters used were: 15 ppm precursor peptide mass error, 0.25 Da fragment mass error, and trypsin specificity with up to two missed cleavages. Variable modifications included Acetyl (Protein N-term), pyroglutamisation (peptide N-terminal Q), carbamidomethylation (C), mono-oxidation (M), phosphorylation (STY) and a custom modification corresponding to the exact mass of the HSP72-NBD nucleotide-competitive TCI **8** (CK). MS1 peak areas were extracted from the LC-MS ion chromatograms using the Thermo Xcalibur Qual Browser 2.2 software.

HSP72 Intact-Protein Mass Spectrometry Data

C17A HSP72-NBD + 200 μM TCI (8)

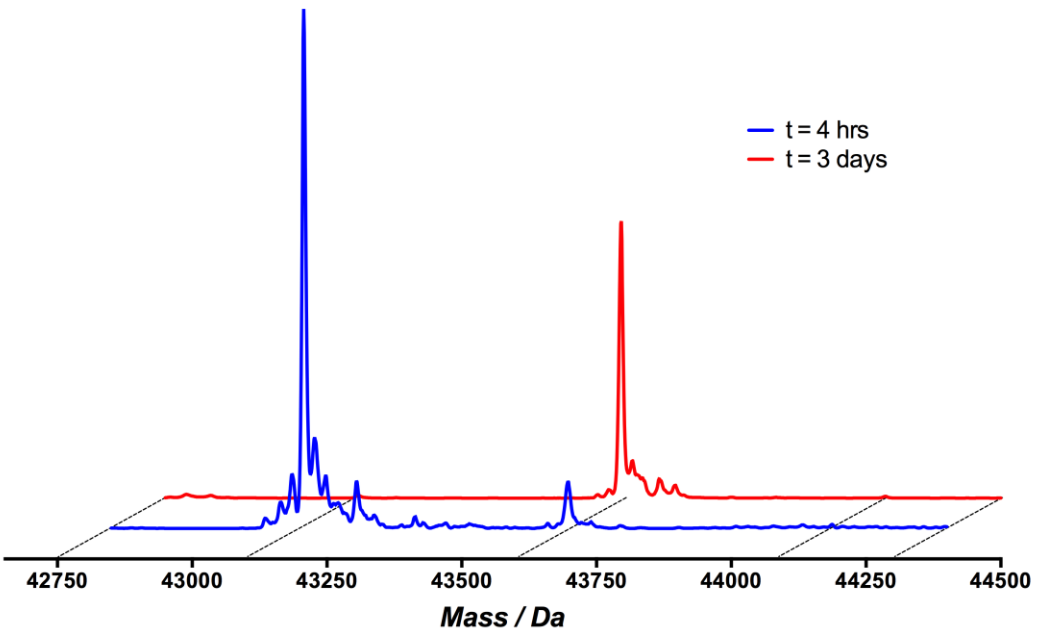

*Intact-protein mass spec showing time-dependent tagging of C17A HSP72-NBD with TCI **8**.*

C267A HSP72-NBD + 200 μM TCI (8)

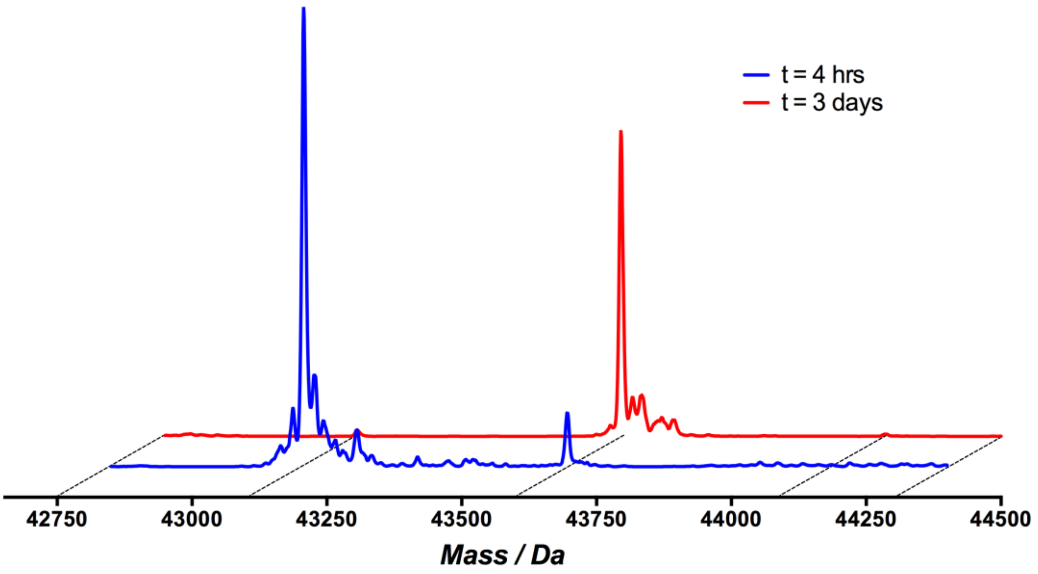

*Intact-protein mass spec showing time-dependent tagging of C267A HSP72-NBD with TCI **8**.*

C306A HSP72-NBD + 200 µM TCI (8)

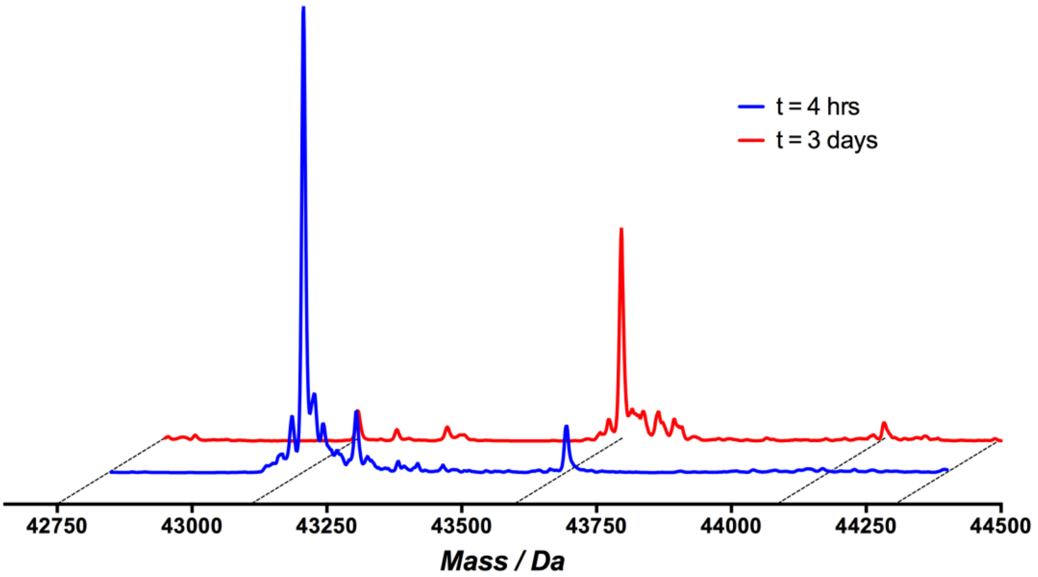

*Intact-protein mass spec showing time-dependent tagging of C306A HSP72-NBD with TCI **8**.*

K56A HSP72-NBD + 200 µM TCI (8)

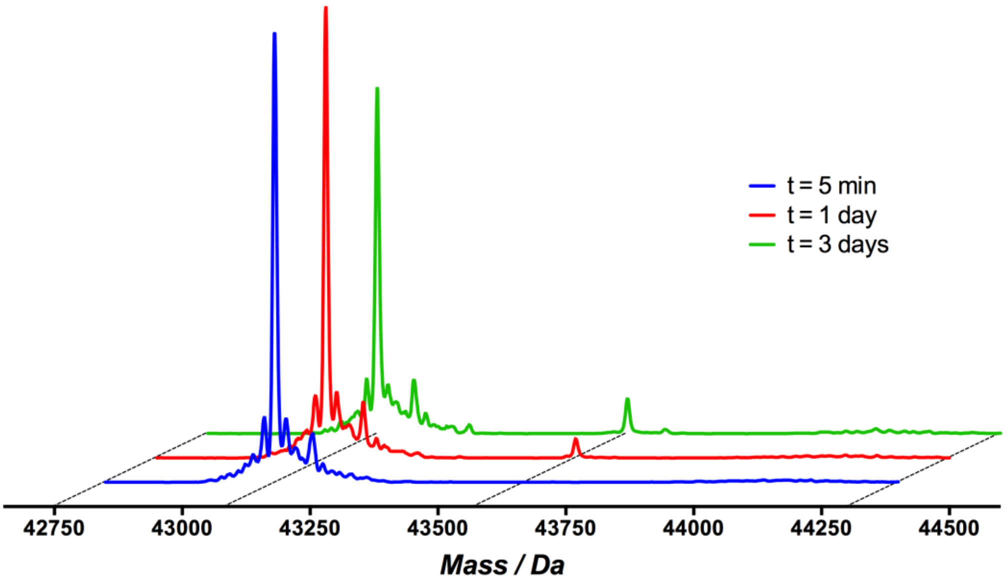

*Intact-protein mass spec showing no significant tagging of K56A HSP72-NBD with TCI **8**. Very small amount of nonspecific tagging is present at 43570 Da.*

WT HSP72-NBD + 200 μM irreversible control (10)

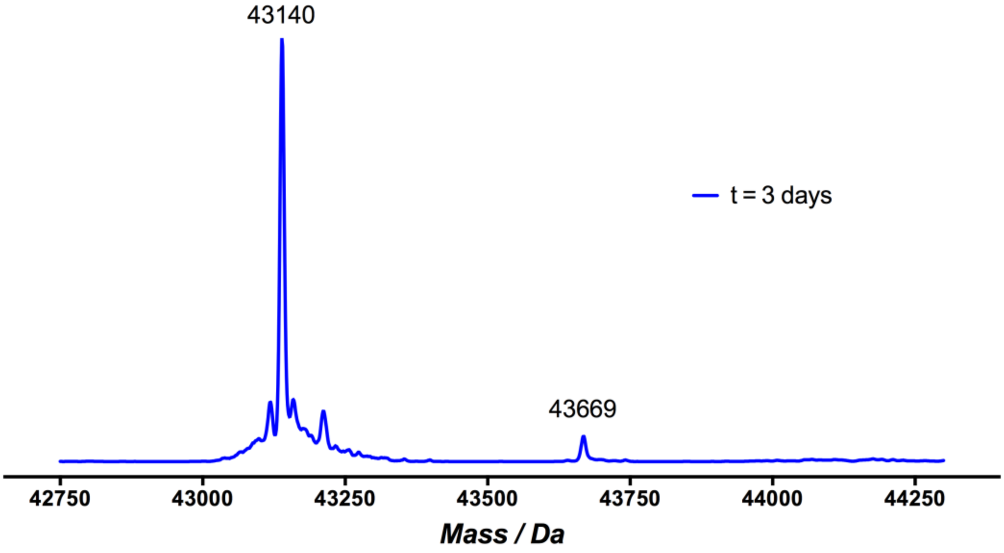

*Intact-protein mass spec showing no significant tagging of WT HSP72-NBD with reactive acetonide control compound **10**. A very small amount of nonspecific tagging is present at 43669 Da.*

WT HSP72-NBD + 200 μM methyl acrylate (S6)

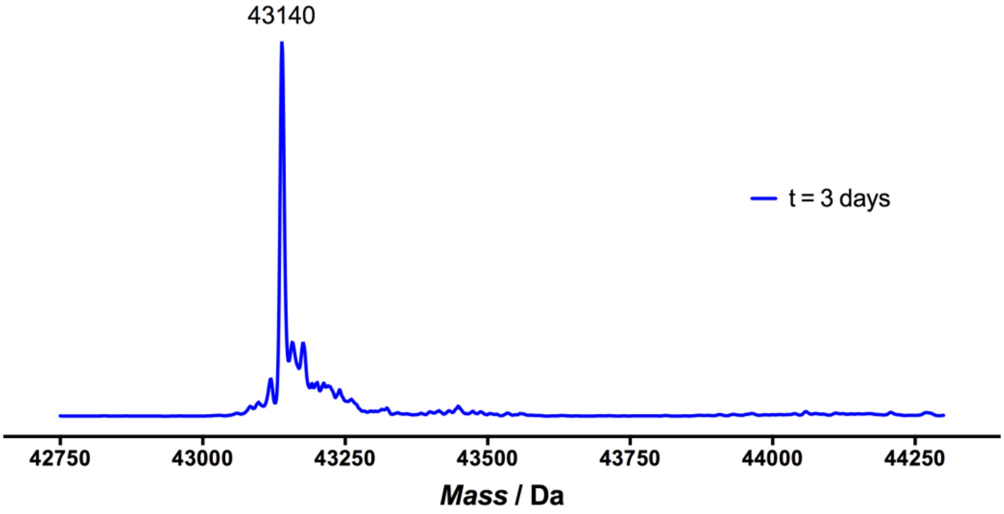

*Intact-protein mass spec showing no tagging of WT HSP72-NBD with methyl acrylate **S6**.*

WT HSP72-NBD + 200 µM YK5 (2)

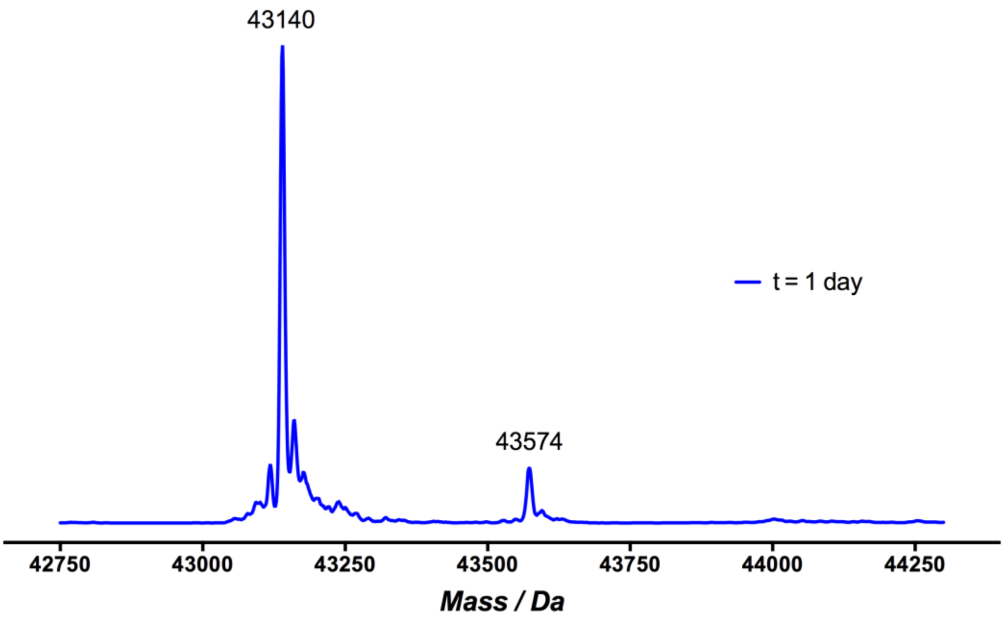

Intact-protein mass spec showing minimal significant tagging of WT HSP72-NBD with tool compound YK5 ***2****.*

WT HSP72-NBD + 200 µM oridonin (3)

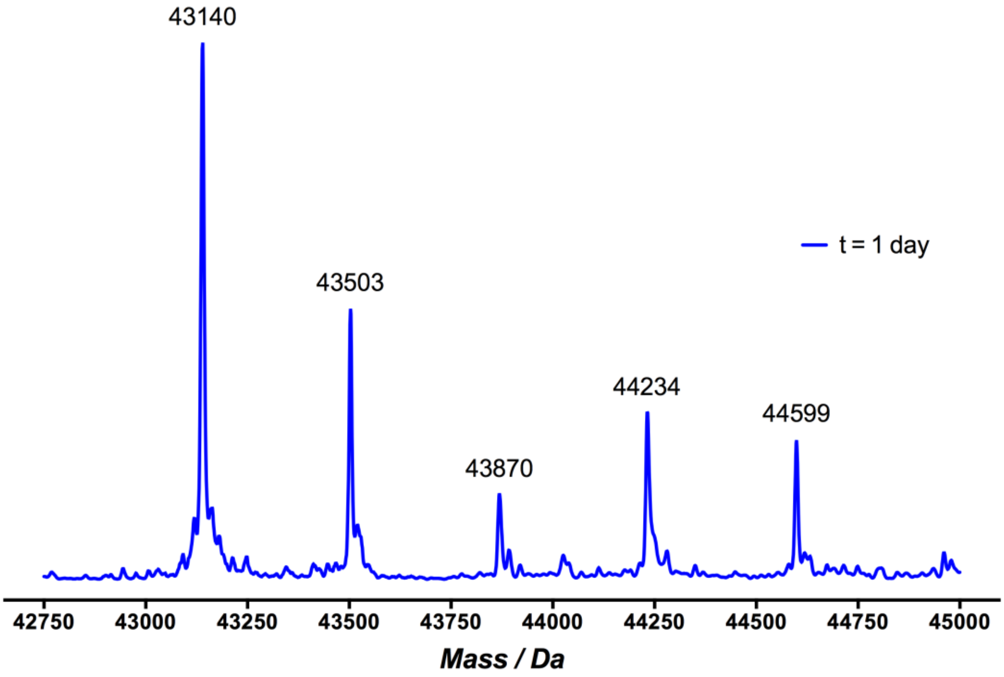

Intact-protein mass spec showing significant nonspecific tagging of WT HSP72-NBD with tool compound oridonin ***3****.*

HSP72 Trypsin-Digest Mass Spectrometry Data

Trypsin-digest data for modification of HSP72-NBD Cys267 with TCI 8

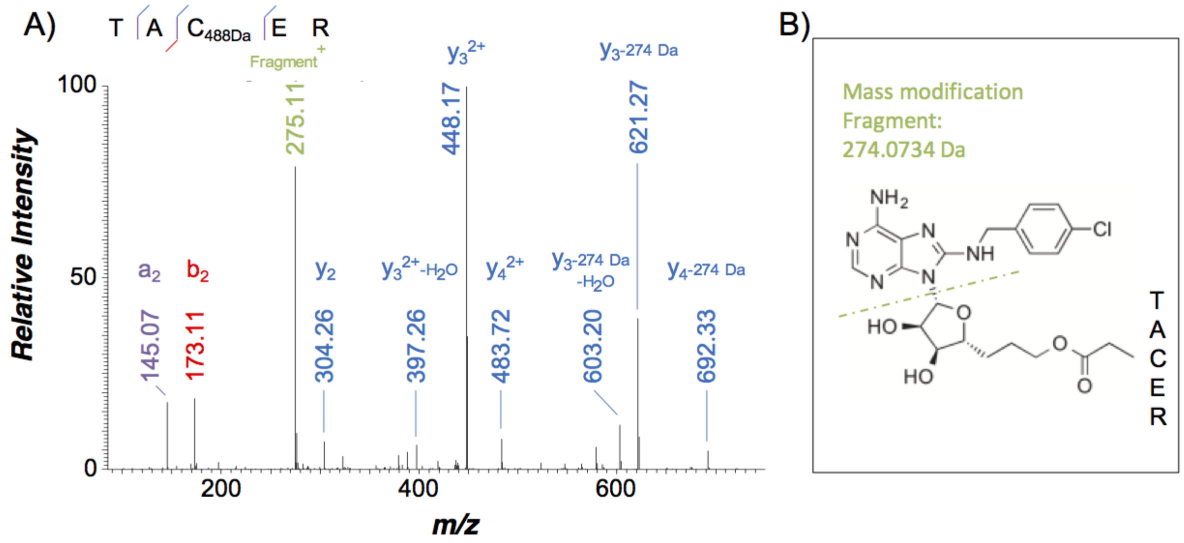

During MS2, fragmentation of the glycosidic bond of TCI **8** occurs, making automated data assignment challenging. This fragment is observed in the spectrum, shown in green (m/z 275.11).

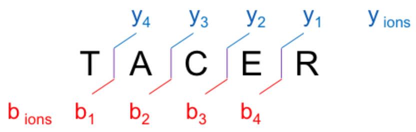

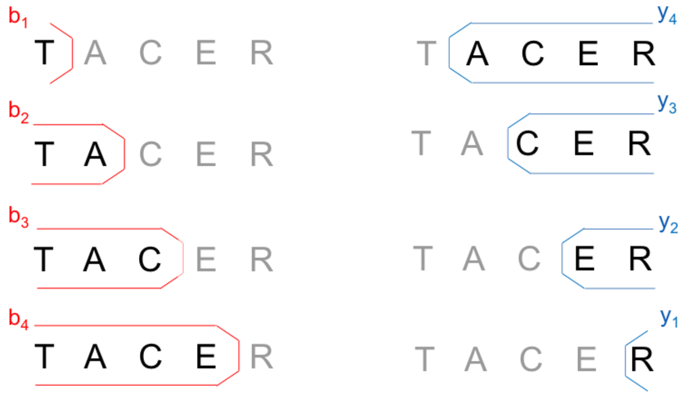

Labelling of MS2 fragmented peaks. The MS2 fragmentation produces b and their respective y ions; a ions are produced from internal fragmentation.

Full MS1 spectra for Trypsin-digested TCI 8-modified HSP72-NBD

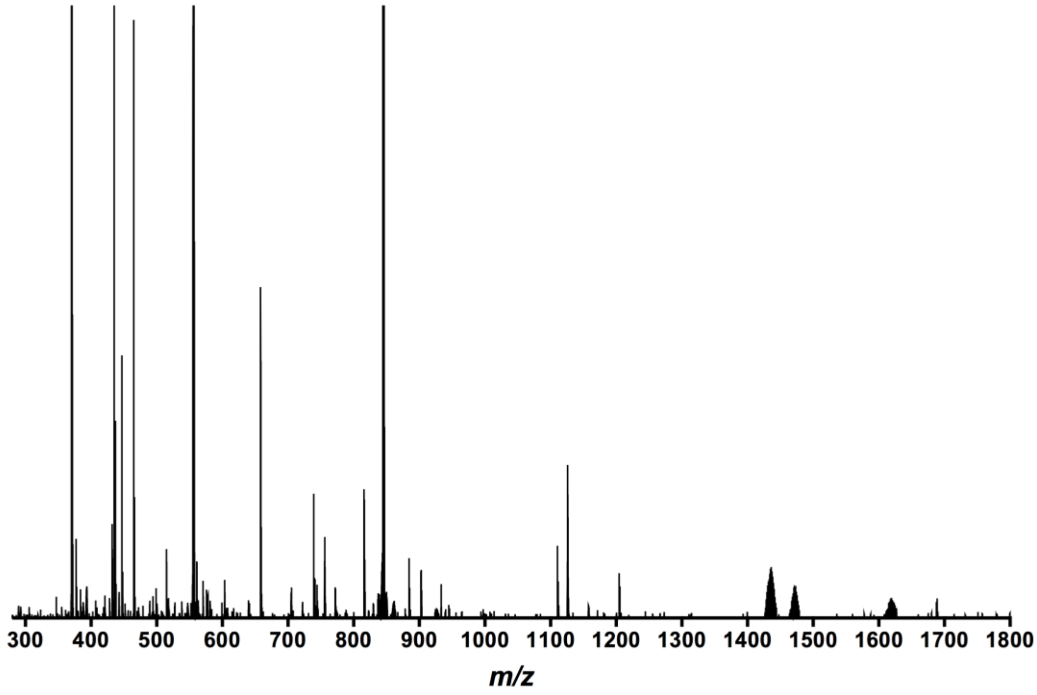

MS1 spectra for Trypsin-digested HSP72-NBD, showing TCI 8-modified 22mer Leu50-Lys71

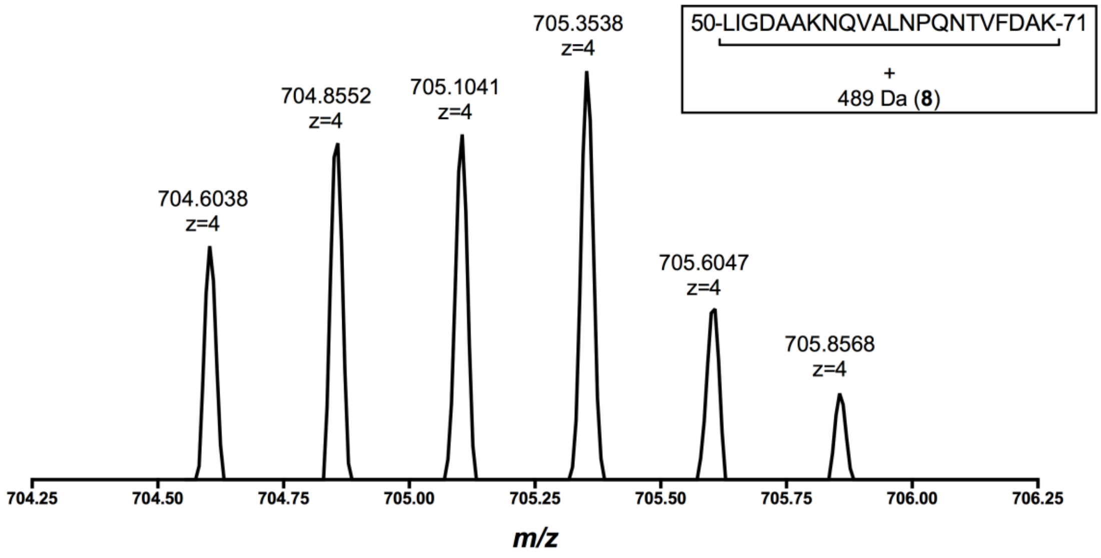

HSP70 Protein Crystal Structure Analysis

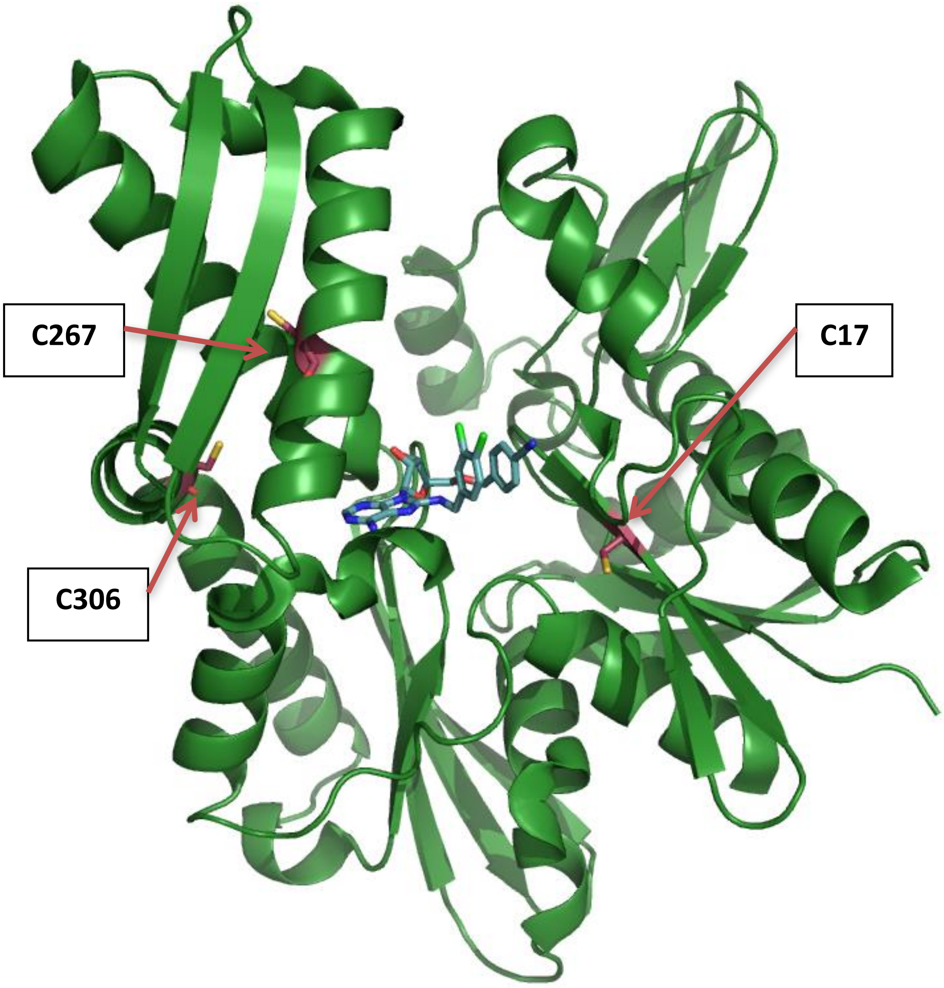

HSP72-NBD bound to nucleotide-competitive inhibitor and cysteine residues
PDB: 4IO8, residues 3 to 379. Cysteine residues are shown in pink. C17 sits at the bottom of the TRI binding site. C267 and C306 are not solvent exposed or accessible.

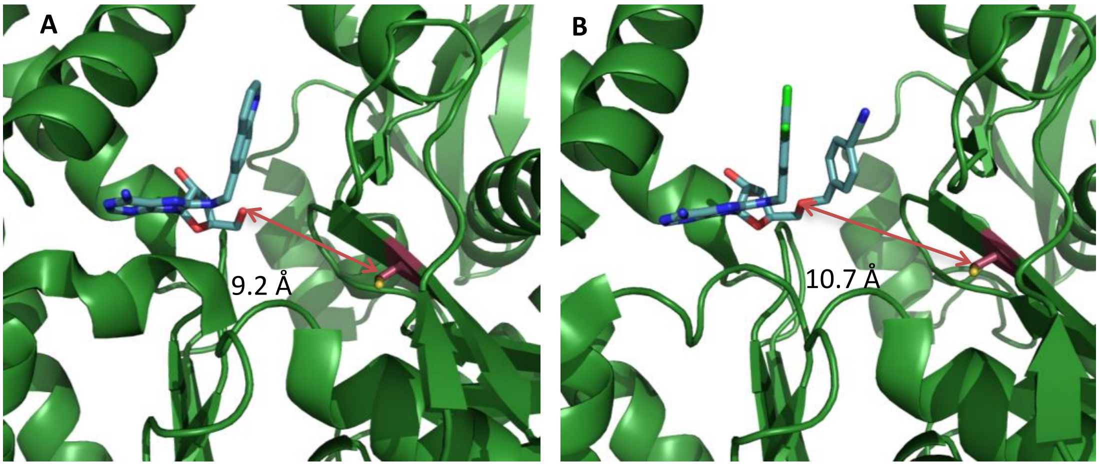

Crystal structures showing conformational flexibility of HSP72-NBD
Distances are between 5'-O of ligand and Cys C-β A) PDB: 5AR0, residues 3 to 383. Closed conformation B) PDB: 3FZL, residues 5 to 381. HSC70-NBD/truncated-Bag1 conformation.

HSP72-NBD Protein Crystallography Experimental

Purified HSP72-NBD was thawed, buffer exchanged into fresh 100 mM HEPES pH 7.5, concentrated to 5 mg/mL and then incubated with 5 mM TCI **8** for 3 days at 4 °C. Co-crystals were grown at 18 °C in sitting drops, using the PEG/ION HT screening plate (Hampton Research). An equal volume of protein and precipitant were used. Crystals typically grew overnight, and 2 high quality rod-like crystals (conditions = PDB: 5MKR, 16% w/v PEG-3350, 0.06 M citric acid, 0.04 M BIS-TRIS propane, pH 4.1. PDB: 5MKS, 16% w/v PEG-3350, 0.02 M citric acid, 0.08 M BIS-TRIS propane, pH 8.8) were fished and cryoprotected with paratone-*N* (Hampton Research) before flash cooling in liquid nitrogen. X-ray diffraction data were collected in-house on a Rigaku FRX with Pilatus 300 K detector. Data were integrated with XDS.[2] All data were imported to MTZ format with POINTLESS,[3] then scaled and merged with AIMLESS[3] and the CCP4 suite.[4] The structures were solved by molecular replacement with PHASER.[5] Structures were refined in iterative cycles of model building with COOT[6] and refinement with BUSTER. TLS groups were selected with PHENIX phenix.find_tls_groups.[7] Ligand restraints were generated with GRADE and MOGUL.[8] The final structure quality was checked with MOLPROBITY.[9] The data collection and refinement statistics are presented below. F_o-F_c electron density figures were generated with CCP4MG.[10] Atomic coordinates and structure factors for the crystal structures of HSP72 with TCI **8** can be accessed using PDB codes 5MKR and 5MKS.

Crystallographic Data Collection and Refinement Statistics

Protein construct	HSP72-NBD 1-380	HSP72-NBD 1-380
Ligand	**8**	**8**
PDB code	5MKR	5MKS
Crystal		
Space group	P 2_1 2_1 2_1	P 2_1 2_1 2_1
Unit cell dimensions (a/b/c in Å)	50.68/86.40/100.23	46.64/83.67/99.21
Unit cell angles (α/β/γ in °)	90/90/90	90/90/90
Data collection and processing		
Beamline	In-house Rigaku	In-house Rigaku
Wavelength (Å)	1.5419	1.5419
Integration program	XDS	XDS
Reduction program	AIMLESS	AIMLESS
Resolution range	32.94 – 1.87	42.67 – 1.99
Number of unique reflections [a]	35583 (3699)	27315 (1866)
Completeness [a]	94.4 (69.4)	99.2 (97.9)
Redundancy [a]	8.9 (2.9)	10.8 (5.3)
R_{merge} (%) [a]	5.1 (23.8)	21.5 (216.7)
I/σ(I) [a]	37.1 (4.8)	7.9 (1.0)
$CC_{1/2}$ [a, b]	0.999 (0.934)	0.991 (0.300)
Refinement		
Program	BUSTER	BUSTER
R_{work} (%)	16.5	21.0
R_{free} (%)	22.3	26.0
Number of residues	386	381
Number of water molecules	772	388
Average B-factor ($Å^2$)	18.0	37.0
Ramachandran favoured (%)	99.17	98.67
Ramachandran outliers (%)	0	0
RMSD bonds (Å)	0.010	0.010
RMSD angles (°)	1.01	1.07

[a] Values in parentheses are for the highest resolution shell.
[b] Half-dataset correlation coefficient, see: Karplus, P. A.; Diederichs, K. Linking crystallographic model and data quality. *Science* **2012**, *336*, 1030−1033.

HSP72 Protein Crystallography Images

Fo-Fc electron density figures for ligand-bound HSP72 structures

A

B

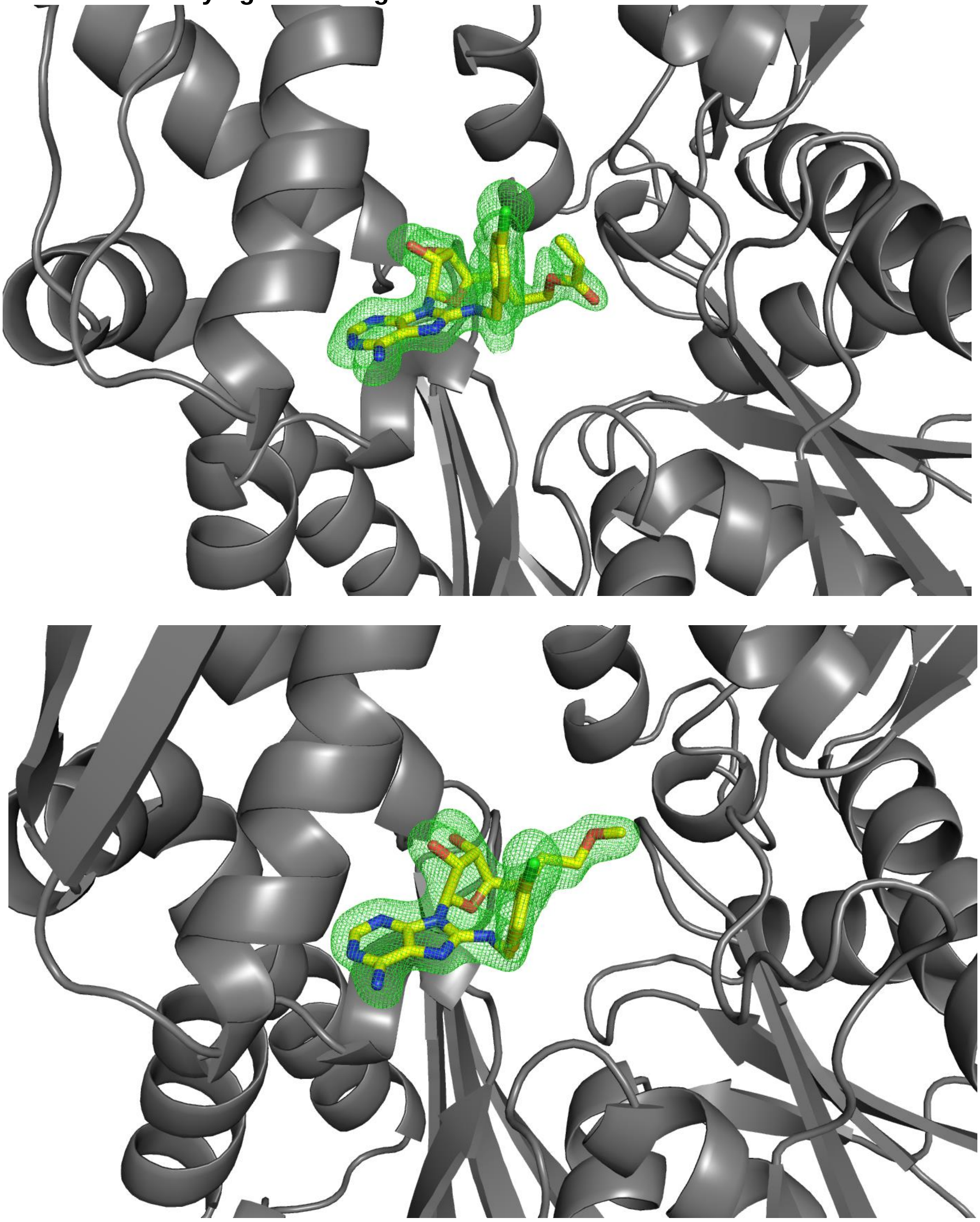

*Fo-Fc electron density maps (green) for HSP72-NBD bound to ligand **8** contoured at 3.5σ. Crystallisation conditions: A) PDB: 5MKR, 16% w/v PEG-3350, 0.06 M citric acid, 0.04 M BIS-TRIS propane, pH 4.1. B) PDB: 5MKS, 16% w/v PEG-3350, 0.02 M citric acid, 0.08 M BIS-TRIS propane, pH 8.8.*

Complete binding contacts of TCI (8)/HSP72-NBD crystal structures

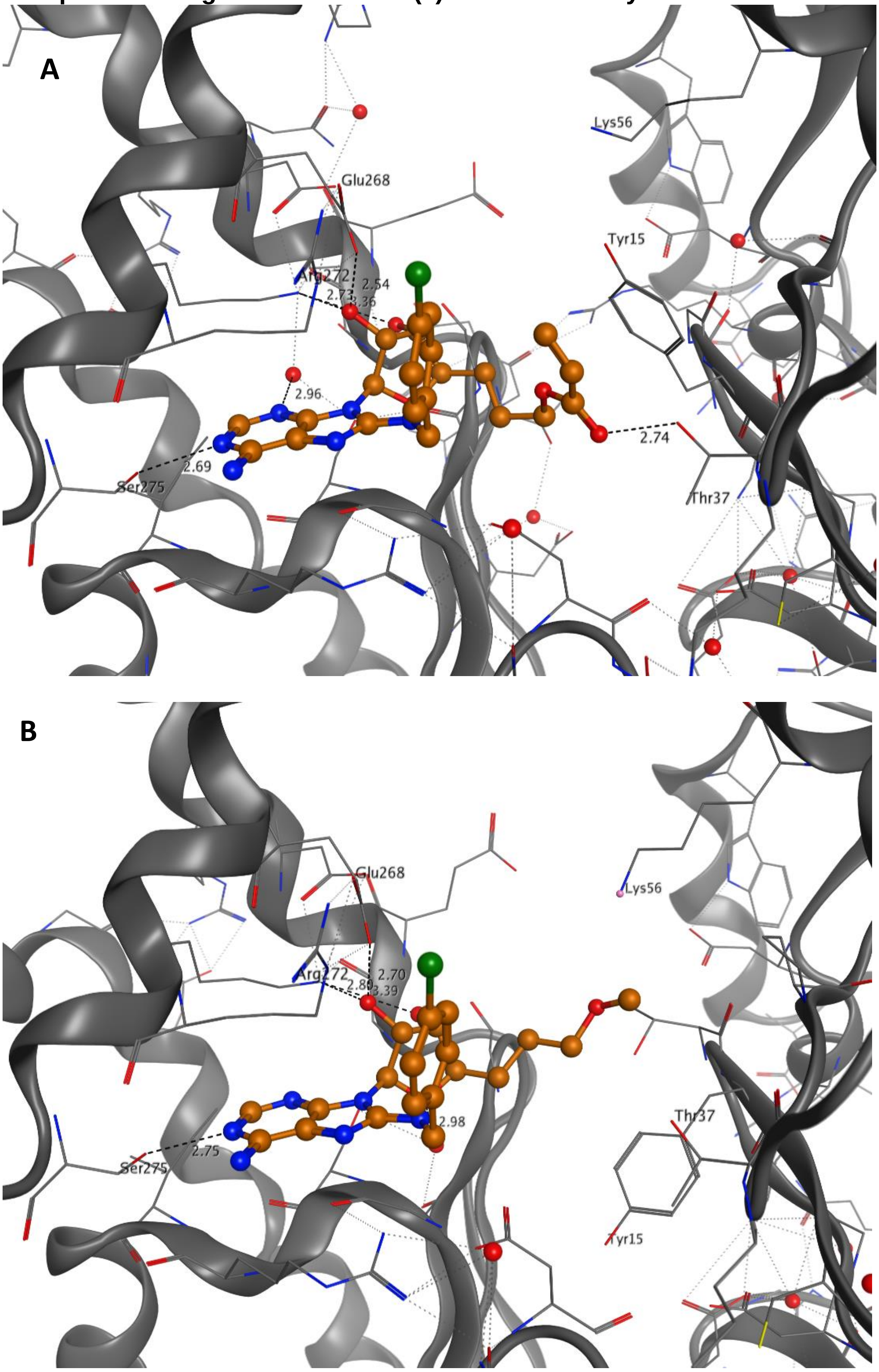

*All distances are in Angstroms. Red spheres represent water molecules. A) All close contacts in TCI **8**/HSP72-NBD co-crystal structure, PDB: 5MKR. B) All close contacts in TCI **8**/HSP72-NBD co-crystal structure, PDB, 5MKS.*

Overlay of TCI (8)/HSP72-NBD co-crystal structures showing alternate conformations

A

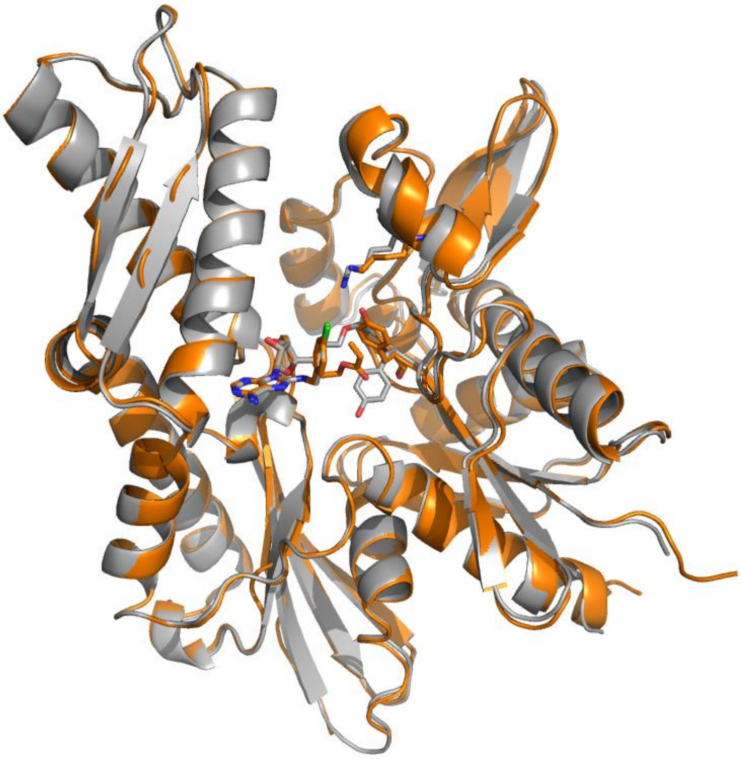

B

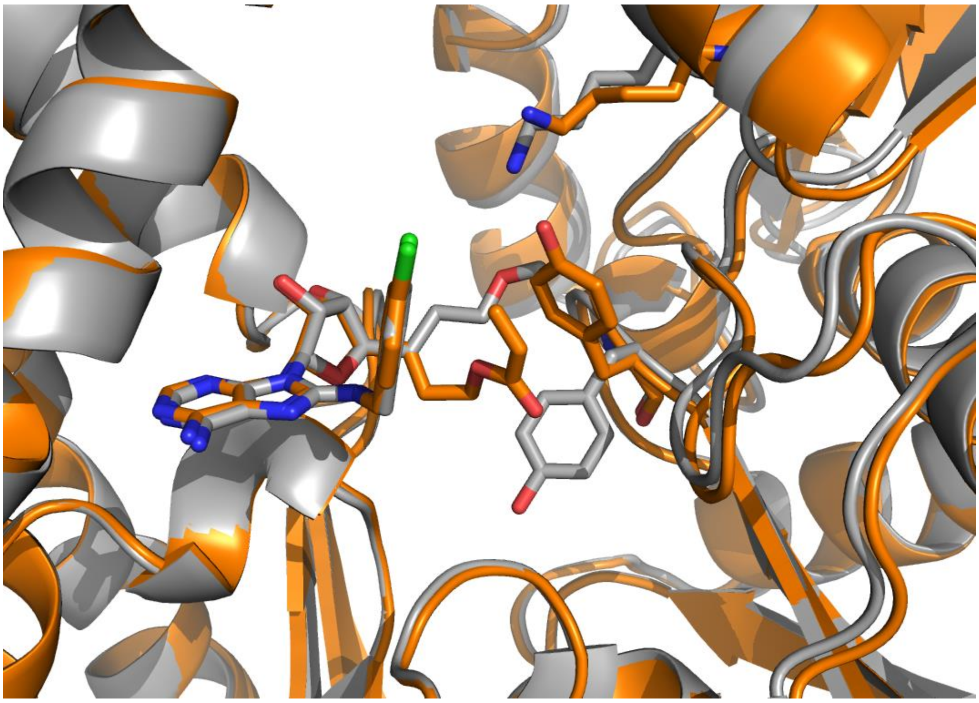

Overlay of Tyr15-up structure (orange) and Tyr15-down structure (grey). A) Whole overlay B) Zoom of overlay showing different conformations.

Structure of HSP72-SBD Covalent Inhibitor Novolactone (S8)

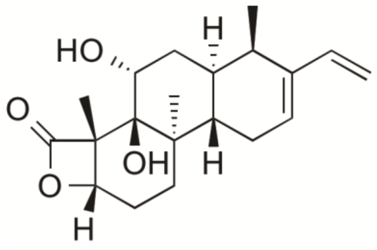

S8

*Novolactone **S8** has been shown by Hassan et al. to modify Glu444 of the HSP72-SBD.*

Structure of HSP72-NBD Reversible Inhibitor Ver-155008 (S9)

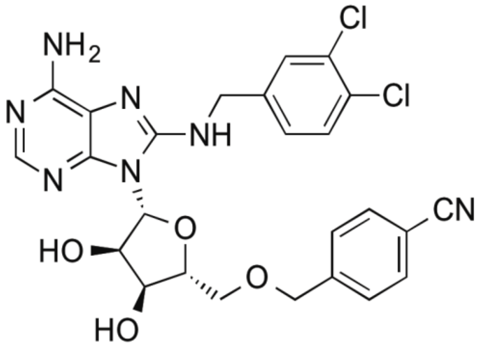

S9

Full Authorship List From Paper References

[4] a) D. S. Williamson, J. Borgognoni, A. Clay, Z. Daniels, P. Dokurno, M. J. Drysdale, N. Foloppe, G. L. Francis, C. J. Graham, R. Howes, A. T. Macias, J. B. Murray, R. Parsons, T. Shaw, A. E. Surgenor, L. Terry, Y. K. Wang, M. Wood, A. J. Massey, *J. Med. Chem.* **2009**, *52*, 1510-1513. b) R. Schlecht, S. R. Scholz, H. Dahmen, A. Wegener, C. Sirrenberg, D. Musil, J. Bomke, H. M. Eggenweiler, M. P. Mayer, B. Bukau, *PLoS One* **2013**, 8, e78443

[9] M. D. Cheeseman, I. M. Westwood, O. Barbeau, M. Rowlands, S. Dobson, A. M. Jones, F. Jeganathan, R. Burke, N. Kadi, P. Workman, I. Collins, R. L. van Montfort, K. Jones, *J. Med. Chem.* **2016**, *59*, 4625-4636

[10] A. Rodina, P. D. Patel, Y. L. Kang, Y. Patel, I. Baaklini, M. J. H. Wong, T. Taldone, P. R. Yan, C. H. Yang, R. Maharaj, A. Gozman, M. R. Patel, H. J. Patel, W. Chirico, H. Erdjument-Bromage, T. T. Talele, J. C. Young, G. Chiosis, *Chem. Biol.* **2013**, *20*, 1469-1480.

[12] A. Q. Hassan, C. A. Kirby, W. L. Zhou, T. Schuhmann, R. Kityk, D. R. Kipp, J. Baird, J. Y. Chen, Y. Y. Chen, F. Chung, D. Hoepfner, N. R. Movva, R. Pagliarini, F. Petersen, C. Quinn, D. Quinn, R. Riedl, E. K. Schmitt, A. Schitter, T. Stams, C. Studer, P. D. Fortin, M. P. Mayer, H. Sadlish, *Chem. Biol.* **2015**, *22*, 87-97.

[14] M. E. Flanagan, J. A. Abramite, D. P. Anderson, A. Aulabaugh, U. P. Dahal, A. M. Gilbert, C. Li, J. Montgomery, S. R. Oppenheimer, T. Ryder, B. P. Schuff, D. P. Uccello, G. S. Walker, Y. Wu, M. F. Brown, J. M. Chen, M. M. Hayward, M. C. Noe, R. S. Obach, L. Philippe, V. Shanmugasundaram, M. J. Shapiro, J. Starr, J. Stroh, Y. Che, *J. Med. Chem.* **2014**, 57, 10072-10079.

[19] a) E. Anscombe, E. Meschini, R. Mora-Vidal, M. P. Martin, D. Staunton, M. Geitmann, U. H. Danielson, W. A. Stanley, L. Z. Wang, T. Reuillon, B. T. Golding, C. Cano, D. R. Newell, M. E. M. Noble, S. R. Wedge, J. A. Endicott, R. J. Griffin, *Chem. Biol.* **2015**, *22*, 1159-1164. b) G. Akcay, M. A. Belmonte, B. Aquila, C. Chuaqui, A. W. Hird, M. L. Lamb, P. B. Rawlins, N. Su, S. Tentarelli, N. P. Grimster, Q. Su, *Nat. Chem. Biol.* **2016**, *12*, 931-936. c) H. P. Morgan, M. J. Walsh, E. A. Blackburn, M. A. Wear, M. B. Boxer, M. Shen, H. Veith, I. W. McNae, M. W. Nowicki, P. A. Michels, D. S. Auld, L. A. Fothergill-Gilmore, M. D. Walkinshaw, *Biochem. J.* **2012**, *448*, 67-72. d) U. P. Dahal, A. M. Gilbert, R. S. Obach, M. E. Flanagan, J. M. Chen, C. Garcia-Irizarry, J. T. Starr, B. Schuff, D. P. Uccello, J. A. Young, *MedChemComm* **2016**, *7*, 864-872.

References

[1] H. Hahne, F. Pachl, B. Ruprecht, S. K. Maier, S. Klaeger, D. Helm, G. Medard, M. Wilm, S. Lemeer, B. Kuster, *Nat. Methods* **2013**, *10*, 989-991.

[2] W. Kabsch, *Acta Crystallogr., Sect. D: Biol. Crystallogr.* **2010**, *66*, 133-144.

[3] P. Evans, *Acta Crystallogr., Sect. D: Biol. Crystallogr.* **2006**, *62*, 72-82.

[4] M. D. Winn, C. C. Ballard, K. D. Cowtan, E. J. Dodson, P. Emsley, P. R. Evans, R. M. Keegan, E. B. Krissinel, A. G. Leslie, A. McCoy, S. J. McNicholas, G. N. Murshudov, N. S. Pannu, E. A. Potterton, H. R. Powell, R. J. Read, A. Vagin, K. S. Wilson, *Acta Crystallogr., Sect. D: Biol. Crystallogr.* **2011**, *67*, 235-242.

[5] A. J. McCoy, R. W. Grosse-Kunstleve, P. D. Adams, M. D. Winn, L. C. Storoni, R. J. Read, *J. Appl. Crystallogr.* **2007**, *40*, 658-674.

[6] P. Emsley, K. Cowtan, *Acta Crystallogr., Sect. D: Biol. Crystallogr.* **2004**, *60*, 2126-2132.

[7] P. D. Adams, P. V. Afonine, G. Bunkoczi, V. B. Chen, I. W. Davis, N. Echols, J. J. Headd, L. W. Hung, G. J. Kapral, R. W. Grosse-Kunstleve, A. J. McCoy, N. W. Moriarty, R. Oeffner, R. J. Read, D. C. Richardson, J. S. Richardson, T. C. Terwilliger, P. H. Zwart, *Acta Crystallogr., Sect. D: Biol. Crystallogr.* **2010**, *66*, 213-221.

[8] I. J. Bruno, J. C. Cole, M. Kessler, J. Luo, W. D. Motherwell, L. H. Purkis, B. R. Smith, R. Taylor, R. I. Cooper, S. E. Harris, A. G. Orpen, *J. Chem. Inf. Comput. Sci.* **2004**, *44*, 2133-2144.

[9] I. W. Davis, A. Leaver-Fay, V. B. Chen, J. N. Block, G. J. Kapral, X. Wang, L. W. Murray, W. B. Arendall, 3rd, J. Snoeyink, J. S. Richardson, D. C. Richardson, *Nucleic Acids Res.* **2007**, *35*, W375-383.

[10] E. Potterton, S. McNicholas, E. Krissinel, K. Cowtan, M. Noble, *Acta Crystallogr., Sect. D: Biol. Crystallogr.* **2002**, *58*, 1955-1957.
